# Sustainability and Time Trends in Electronic Patient-Reported Outcome Assessment in Routine Cancer Care: Systematic Scoping Review and Follow-Up Survey

**DOI:** 10.2196/69398

**Published:** 2025-04-25

**Authors:** Niclas J Hubel, Samuel M Vorbach, Kelly M de Ligt, Ines S Rathgeber, Katharina Beyer, Lisa M Wintner, Barbara Faller, Jasmin Nemec, Bernhard Holzner, Monika Sztankay, Jens Lehmann

**Affiliations:** 1 University Hospital of Psychiatry II Medical University of Innsbruck Innsbruck Austria; 2 Department of Radiation Oncology Medical University of Innsbruck Innsbruck Austria; 3 Department of Psychosocial Research and Epidemiology Netherlands Cancer Institute Amsterdam The Netherlands; 4 Department of Urology ​​Erasmus Medical Center Cancer Institute University Medical Center Rotterdam Rotterdam The Netherlands; 5 Department of Psychology Universität Innsbruck Innsbruck Austria

**Keywords:** patient-reported outcome measures, cancer, clinical routine, health-related quality of life, quality of care, mobile applications, digital technology

## Abstract

**Background:**

Routine electronic assessment of patient-reported outcomes (ePROs) can improve cancer care; yet, its implementation in routine practice and long-term sustainability remain unclear. Understanding these aspects is critical to advancing the field.

**Objective:**

To review and describe the past and current status, time trends, and long-term sustainability of clinical ePRO applications in routine oncology care.

**Methods:**

We conducted a systematic review of publications on ePRO use in oncology care up to December 31, 2023, searching PubMed and Web of Science and extracting data on clinical ePRO applications. We included peer-reviewed studies including patients with cancer using ePRO assessments in clinical practice, excluding research letters and conference abstracts. Data from the review were analyzed using descriptive statistics and univariate regression models to evaluate time trends, with year of publication as the predictor. A follow-up survey was sent to authors of published ePRO applications to assess their current use of the application or reasons for discontinuation. Responses from the follow-up survey were analyzed descriptively.

**Results:**

For the review, we screened 2933 references, and 303 met inclusion criteria. Results showed that Europe was the most common region (n=141, 46.5%), and study populations consisted mostly of adult patients (n=276, 91.1%) under chemotherapy treatment (n=124, 40.9%) assessed in an outpatient setting (n=261, 86.1%). The EORTC (European Organisation for Research and Treatment of Cancer; n=77, 25.4%) and PRO-CTCAE (Patient-Reported Outcomes Version of the Common Terminology Criteria for Adverse Events) questionnaires (n=65, 21.5%) were most frequently integrated into ePRO applications. In the univariate analysis, we found that publications increased significantly over time (2003-2023, *P*<.001). Trends showed a rise in mobile app use (odds ratio [OR] 1.211, *P*<.001), remote assessments (OR 1.094, *P*=.002), and feedback provided to patients (OR 1.060, *P*=.04). Of the 303 studies, 221 unique clinical ePRO applications were identified, merging publications at the application level. The follow-up survey had a 35.3% response rate (78/221), with 61.1% of ePRO applications still in use, lasting a median of 5 years. The most common reason for discontinuation was a lack of funding and resources (42.9%, 12/28).

**Conclusions:**

The field of ePRO assessment in oncology is rapidly evolving, with a shift toward remote, app-based tools and a growing emphasis on providing feedback to patients. We present, for the first time, data on the sustainability of ePRO use in routine care. While our findings offer valuable insights, they should be interpreted in light of potential response bias in the follow-up survey. Several ePRO applications remain in active use, highlighting potential for long-term integration into clinical practice. However, financial constraints, limited reimbursement models, and challenges with workflow integration continue to hinder broader and more sustainable adoption. Addressing these barriers will be essential to support the continued use of ePROs in clinical care.

## Introduction

Adequate symptom monitoring is a cornerstone of clinical care for patients with cancer. During and after treatment, patients frequently experience symptoms that can impact their health-related quality of life (HRQOL), therapy compliance, or even be life-threatening [1-3]. Routine assessment and use of patient-reported outcomes (PROs) in clinical care can improve care for patients with cancer, resulting in better HRQOL, reduced hospitalization rates, and even prolonged survival [1,4-9]. Accordingly, the European Society for Medical Oncology advises the routine assessment of PROs in clinical care, emphasizing the importance of electronic PRO (ePRO) assessments [10].

In recent years, electronic approaches to collecting PRO data—commonly referred to as ePRO assessments—have gained increasing importance. Real-time digital platforms (hereafter referred to as ePRO software) offer an efficient and scalable solution to systematically collect, store, and report PRO data in clinical settings. Compared to traditional paper-based methods, ePRO software facilitates standardized and timely assessments, can potentially reduce response burden and missing data, and enhance overall usability and patient satisfaction [7,11-14].

Despite all these advantages, there seems to be a challenge to sustainably implement such ePRO interventions into routine workflows [15,16]. The complex nature of routine care, fragmented workflows, limited resources, and reluctance to change within health care systems may contribute to the slow progress in implementing ePRO software tools outside of research settings [16-18]. Another common difficulty lies in obtaining reliable data on the use of ePRO software in clinical practice settings [19]. While many studies are published during the initial development and implementation phase [20], there is little information on the long-term use of those *clinical ePRO applications* (referring to the use of ePRO software in a specific clinical context). A considerable portion of developed clinical ePRO applications may be discontinued following the completion of research initiatives, potentially failing to transition into long-term use in clinical care.

The landscape of ePRO assessment in routine oncology is evolving rapidly, with an increasing number of publications emerging. However, since the review by Warrington et al in 2019 (which included studies published until 2017) [21], no recent comprehensive reviews have captured this trend across the field of oncology as a whole—a considerable time span for a fast-changing field. Current reviews on clinical ePRO applications often have limitations that prevent a comprehensive overview of the current landscape of clinical ePRO applications used in oncology practice, like focusing on specific patient populations or applying narrow inclusion criteria (eg, focusing on palliative care applications only or on ePRO use for specific types of treatment) [20,22].

The objectives of this systematic scoping review and follow-up survey are to review and describe the past and current practice of published ePRO use in oncology clinical care; to assess potential trends in the clinical use and assessment of ePRO data over time; and to assess the sustainability and long-term use of the reported clinical ePRO applications.

## Methods

### Review

The review was conducted according to the methodological framework by Arksey and O’Malley [23] and reported according to the PRISMA-ScR (Preferred Reporting Items for Systematic Reviews and Meta-Analyses extension for Scoping Reviews) 2020 checklist (see Methods S1 in Multimedia Appendix 1) [24].

We searched PubMed and Web of Science for studies published until December 31, 2023, without restrictions regarding the publication date. A detailed description of the search strategy is provided in Methods S2 in Multimedia Appendix 1. Reviews and meta-analyses identified in the initial search were used for a backward search (identifying relevant studies based on the cited references).

We included studies that were conducted exclusively with oncology patients, reported on the electronic assessment of PROs, were peer-reviewed primary research, and were available in the English language. We excluded studies that evaluated experimental interventions other than the clinical ePRO application, research letters, editorials, and conference abstracts.

The review software DistillerSR (DistillerSR Inc) [25] was used for the 2-level study selection process and the subsequent data extraction. References were independently screened and reviewed by 2 individual reviewers experienced with ePROs. Disagreements were resolved in discussion. If no agreement was reached, a third (senior) reviewer was consulted. The data extraction followed the same procedure.

For general study characteristics, we extracted details on authors, address of correspondence, year, and country of origin. We categorized the respective studies into the following publication types reflecting different stages of the clinical ePRO application and its use: system development (incomplete system, still under technical evaluation), feasibility study (finalized system, still under practical evaluation), study protocol, trial (evaluating a finalized ePRO system), implementation study (integration of finalized system into clinical workflow), and routine application (describing use of PRO data from an implemented system).

We charted the purpose of ePRO use according to the International Society for Quality of Life Research (ISOQOL) user guide for implementing PROs in clinical practice [26]. Screening tools (one-time PRO assessment with feedback to clinicians), monitoring tools (repeated assessment with feedback of results to clinicians), patient-centered care (feedback of PRO results to patients), ePRO assessment without direct feedback, decision aids (information about treatment options or similar), evaluating quality of care, and facilitating multidisciplinary team communications. Patient groups were classified into inpatients and outpatients, and the time of PRO assessment was determined according to the respective care phase.

### Review: Analysis

We report descriptive statistics of the publication characteristics and all extracted variables. All missing data were classified as “not reported” and excluded from all analyses. To investigate time trends in ePRO data capture over time, we calculated binomial Poisson and logistic regressions with the year of publication as the predictor and the number of publications per year as the outcome. Further, we conducted univariate logistic regressions with the year of publication as the predictor and one of the following selected variables as the criterion: inpatient population, outpatient population, remote assessment, in-hospital assessment, use of website, use of app, provision of PRO feedback to patients, provision of PRO feedback to clinicians, internal PRO software development, or external PRO software development. These variables were selected as markers for overall shifts within the field and indicators of technological advancements in the last 20 years. They reflect evolving approaches to data collection and to the integration of digital tools in clinical care.

The selected outcomes were coded as 1=[true] and 0=[false]. Model coefficients are expressed as odds ratios (ORs) for the logistic regressions and as risk ratios for the Poisson regression, each with 95% CIs and an α level of .05. Calculations were done using R (version 4.3.1; R Core Team) [27].

### Follow-Up Survey

We sent a follow-up questionnaire to the corresponding authors of all clinical ePRO applications that were identified based on the publications in the review (ie, merging publications on the distinct use of an ePRO software in a specific clinical context). The questionnaire was distributed via email, and up to 2 reminders were sent if needed. We developed the questionnaire among the authors who are experienced with the implementation of ePROs and pilot-tested it internally before distribution.

### Follow-Up Survey: Measures

The survey aimed to evaluate the status of clinical ePRO application use. Participants were asked whether the application was still in use, with follow-up questions tailored accordingly: reasons for discontinuation or details about current usage practices were explored in alignment with the ISOQOL user guide [26]. Nonusers were invited to share what changes or features would encourage them to consider adopting ePRO assessments or systems in the future.

Additional questions addressed the duration of ePRO application use, the average number of participating patients per month, and satisfaction levels, which were rated on a 4-point scale. Participants also had the opportunity to provide suggestions for improvement through an open-text field. The complete survey is detailed in Methods S3 in Multimedia Appendix 1.

### Follow-Up Survey: Analysis

We report descriptive statistics of all variables; missing data were classified as “not reported” and excluded from all analyses. To evaluate potential response bias within our data, we calculated differences in the distribution of the respondents to our survey using Chi-squared tests and univariate logistic regressions for the following variables: the year of the last publication related to the respective ePRO clinical application, the total number of published articles referring to the clinical ePRO application, the region of the cancer centers, and the publication type of the last article published based on the clinical ePRO application.

Calculations were done using R (version 4.3.1) [27].

## Results

### Review

**Figure 1:**
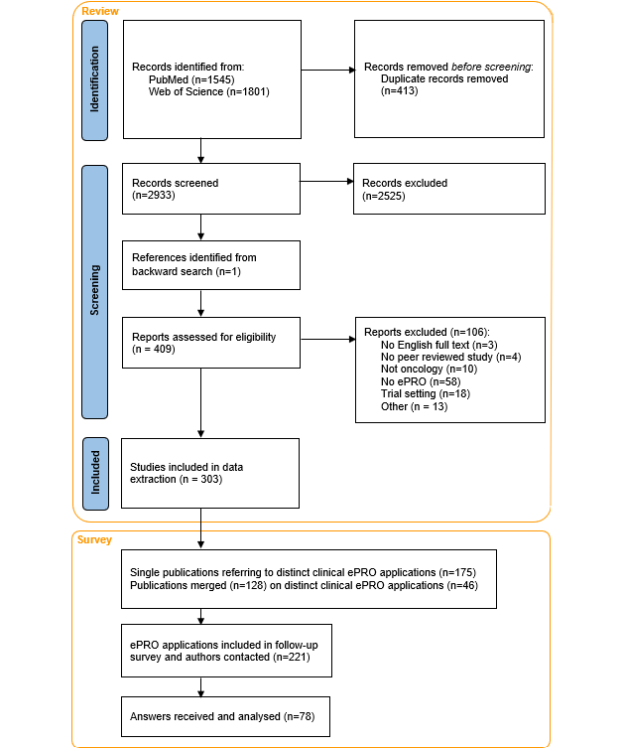
PRISMA (Preferred Reporting Items for Systematic Reviews and Meta-Analyses) flowchart. ePRO: electronic patient-reported outcome.

The initial literature search yielded 3346 references. After the removal of 413 duplicates, the remaining 2933 records underwent title abstract screening for eligibility, of which 409 studies were included in the full-text review. A final 303 studies were included in the review. Figure 1 depicts the PRISMA-ScR flowchart. The complete list of references can be found in eReferences in Multimedia Appendix 1. The main reasons for exclusions were that the full text did not include a clinical ePRO application (n=58) and alternative interventions other than ePRO monitoring were examined (n=18). Publication dates ranged from 2003 until 2023.

Based on the 303 publications included in the review, we identified 221 unique clinical ePRO applications. Most clinical ePRO applications (175, 57.8%) were reported only in a single publication, while 46 clinical ePRO applications were reported in multiple publications (median 2, IQR 1-1, range 2-10).

### Study Characteristics

The majority of included studies were conducted in Europe (46.5%) and North America (39.6%), with the United States being the most common country (34.3%; Table 1, and Table S1 in Multimedia Appendix 1). According to our classification, the most used study types were ePRO feasibility studies (33%) and studies describing the routine use of clinical ePRO applications in clinical practice (17.8%). The complete study data are shown in Multimedia Appendix 2.

**Table 1:** Study characteristics (N=303).

		Publications	
		Number	Percentage
**Region^a^**			
	Europe	141	46.5
	North America	120	39.6
	Asia	22	7.3
	Australia	12	4.0
	South America	4	1.3
	International	1	0.3
	Not reported	3	1.0
**Publication types**			
	Feasibility study	100	33.0
	Routine application	54	17.8
	System development	42	13.9
	Trial	41	13.5
	Study protocol	40	13.2
	Implementation study	26	8.6
**Patient group**			
	Outpatients	261	86.1
	Inpatients	10	3.3
	Both	18	5.9
	Not reported	14	4.6
**Patient age groups**			
	Adults	276	91.1
	Children and adolescents (<18 years)	13	4.3
	Not reported	14	4.6
**Cancer type**			
	Not specified or defined	110	36.3
	Application used in specific cancer types (multiple could apply)	193	63.7
	Breast	78	40.4
	Gastrointestinal	37	19.2
	Lung	37	19.2
	Prostate	34	17.6
	Colorectal	31	16.1
	Head and neck	26	13.5
	Hematological	25	13.0
	Gynecological	17	8.8
	Other^b^	15	7.8
**Treatment (multiple could apply)**			
	Chemotherapy	124	40.9
	Radiotherapy	64	21.1
	Surgery	56	18.5
	Hormonal therapy	31	10.2
	Targeted therapy	24	7.9
	Immunotherapy	23	7.6
	No active treatment	21	6.9
	Other^c^	6	2.0
	Not reported	90	29.7

^a^See Table S1 in Multimedia Appendix 1 for full list.

^b^Bladder, central nervous system, liver, neuroendocrine, renal, retinoblastoma, thyroid, and genitourinary.

^c^Active surveillance, cryotherapy, and stem cell transplantation.

The applications were most frequently designed for patients with breast (n=78, 25.7%), gastrointestinal (n=37, 12.2%), and lung (n=37, 12.2%) cancers. Most studies were conducted with adult patients (n=276, 91.1%), and among outpatients (n=261, 86.1%) treated with chemotherapy (n=124, 40.9%).

### Reported use of ePRO Assessment in Clinical Practice

The most common use cases of ePROs, according to the ISOQOL classification, were as monitoring tools (n=219, 72.3%) and for patient-centered care (n=123, 40.6%; Table 2). Physicians (n=166, 54.8%) and nurses (n=154, 50.8%) were the main users of ePRO software, while other health care professionals were rarely mentioned (less than 3%). The user groups were not specified in 30.7% of the publications.

The ePRO assessment was predominantly conducted remotely (n=147, 48.5%) via a website interface (n=122, 40.3%). The respective ePRO software tools were partly developed in-house at the respective cancer centers (n=106, 35%) or by external IT vendors (n=109, 36%). A total of 145 unique ePRO software tools were identified, of which 39 were mentioned in more than 1 publication (median 1 publication, IQR 1-3, range 1-16). In total, 55 (18.2%) publications did not report any distinctive tool (Table S2 in Multimedia Appendix 1 for more information).

The questionnaires most commonly assessed via ePRO were EORTC (European Organisation for Research and Treatment of Cancer) measures (n=77, 25.4%), closely followed by the PRO-CTCAE (Patient-Reported Outcomes Version of the Common Terminology Criteria for Adverse Events) questionnaire (n=65, 21.5%). A full list of the 136 used questionnaires can be found in Table S3 in Multimedia Appendix 1.

**Table 2:** Characteristics and use of ePRO^a^ assessments (N=303).

				Publications			
				Number			Percentage
**Assessment setting**							
	Remote		147			48.5	
	In clinic		79			26.1	
	Both in clinic and remote		55			18.2	
	Not reported		22			7.3	
**Clinical user group (multiple could apply)**							
	Physicians		166			54.8	
	Nurses		154			50.8	
	Psychologists		8			2.6	
	Pharmacists		7			2.3	
	Social workers		6			2.0	
	Dietitians		3			1.0	
	Others (physiotherapists, case managers, etc)		10			3.3	
	Not reported		93			30.7	
**Technology**							
	Website interface		122			40.3	
	App		75			24.8	
	Both, website interface and app		41			13.5	
	Not reported		65			21.5	
**Technical development of the software**							
	External IT vendors		109			36.0	
	In-house		106			35.0	
	Joint development		5			1.7	
	Not reported		83			27.4	
**Time of ePRO assessment (multiple could apply)**							
	During active treatment		213			70.3	
	During early follow-up (<2 years after treatment)		125			41.3	
	During long-term follow-up (≥2 years after treatment)		45			14.9	
	In palliative care		36			11.9	
	Pre-treatment		12			4.0	
	Not reported		31			10.2	
**Purpose of ePRO use (multiple could apply)**							
	Monitoring tools (data collection over time with feedback of results to clinicians)	219			72.3		
	Patient-centered care (data collection with feedback of PRO results to patients)	123			40.6		
	ePRO assessment without feedback	59			19.5		
	Decision aids (for shared decision-making)	10			3.3		
	Evaluating quality of care	8			2.6		
	Screening tools (one-time PRO assessment with feedback to clinicians)	7			2.3		
	Facilitating multidisciplinary team communications		5			1.7	
**PROMs assessed within the ePRO software tool (multiple could apply)**							
	EORTC^b^ questionnaires (C30 and modules)		77			25.4	
	PRO-CTCAE^c^		65			21.5	
	EQ-5D		29			9.6	
	FACIT^d^ questionnaires		27			8.9	
	Own development		27			8.9	
	Others^e^		307			93.4	
	Not reported		34			11.2	

^a^ePRO: electronic patient-reported outcome.

^b^EORTC: European Organisation for Research and Treatment of Cancer.

^c^PRO-CTCAE: Patient-Reported Outcomes Version of the Common Terminology Criteria for Adverse Events.

^d^FACIT: Functional Assessment of Chronic Illness Therapy.

^e^See Table S3 in Multimedia Appendix 1 for full list.

### Time Trends in ePRO Use

The number of publications increased significantly over time (risk ratio 1.20, 95% CI 1.17-1.22, *P*<.001), rising from just 2-4 publications per year in the early 2000s to a peak of 42 in 2023. Notable increases occurred from 2014 onward, with particularly sharp growth between 2015 and 2017 and again after 2019. A detailed annual overview is provided in Multimedia Appendix 3.

The binomial logistic regression model for app use was statistically significant (OR 1.21, 95% CI 1.12-1.32, *P*<.001), meaning that for each additional year, the likelihood that the ePRO assessment was embedded in an app increased by 21.1%. At the same time, fewer publications per year reported the use of a website interface (OR 0.85, 95% CI 0.77-0.93, *P*<.001).

Remote assessments were more frequently reported in recent years (OR 1.09, 95% CI 1.03-1.16, *P*=.002) with an increase of 9.4% per year. The likelihood of articles describing in-clinic assessments decreased by 8% per year (OR 0.92, 95% CI 0.87-0.97, *P*=.004).

Publications over time increasingly described providing patients PRO feedback (increase of 6% per year; OR 1.060, 95% CI 1.00-1.12, *P*=.04). There was no significant change over time in providing PRO feedback to physicians (*P*=.22), but this number was consistently high throughout the years (data not shown). Similarly, no time trend was observed regarding the reported patient population for either inpatient (*P*=.97) or outpatient settings (*P*=.87). The odds of using software tools developed by an external IT vendor increased significantly over time (OR 1.08, 95% CI 1.01-1.15, *P*=.02), whereas the trend for in-house development was not significant (*P*=.06).

### Follow-Up Survey

The follow-up survey was sent out to all corresponding authors and received a response rate of 35.3% (78/221).

There was no link between whether someone responded and the year their last clinical ePRO publication appeared (*P*=.15) or the type of publication (*P*=.13). However, response rates varied significantly between regions (*χ*²_6_=17.0, *P*=.009). Specifically, response rates were lower in Asia (3/20, 15%) and North America (24/90, 26.7%), while they were higher in Australia (5/6, 83.3%), Europe (43/97, 44.3%), and South America (2/4, 50%). Finally, authors who had published more articles on their clinical ePRO application were also more likely to respond (OR 1.44, 95% CI 1.08-2.06, *P*=.02).

Of the 78 responses received, 50 (64.1%) indicated that the respective application was still in use and had been operational for a median of 5 (IQR 3-10) years (Table 3).

The most prevalent use cases, according to the ISOQOL classification, were using the clinical ePRO application as a monitoring tool (34/50, 68%), for patient-centered care (32/50, 64%), and for research purposes (36/50, 72%), with respondents able to select multiple options. Further, respondents reported 1-50 (40%) or 51-200 (30%) average patient users per month.

Among the authors who reported that their ePRO application was no longer in use (28/78, 35.9%), a lack of funding was the most common reason for discontinuation (12/28, 42.9%). About the same number of respondents reported that they would resume the ePRO application in case the required funding or resources (eg, administrative support, dedicated personnel, or IT support) were available (13/28, 46.4%). In total, 8 (28.6%) authors reported that they were no longer using the ePRO application they had been contacted for but that they had switched to different ePRO software.

**Table 3:** Follow-up survey (N=78).

		Publications	
		Number	Percentage
Clinical ePRO^a^ application still in use		50	64.1
**Patients participating per month**			
	1-50	20	40.0
	51-200	15	30.0
	201-500	4	8.0
	>500	10	20.0
	Not reported	1	2.0
**Satisfaction with clinical ePRO application**			
	Not at all	0	0.0
	A little bit	7	14.0
	Quite a bit	23	46.0
	Very much	20	40.0
**Purpose of ePRO use (multiple could apply)**			
	Research	36	72.0
	Monitoring tools (data collection over time with feedback of results to clinicians)	34	68.0
	Patient-centered care (data collection with feedback of PRO results to patients)	32	64.0
	Screening tools (one-time PRO assessment with feedback to clinician)	17	34.0
	Facilitating multidisciplinary team communications	17	34.0
	Decision aids (for shared decision-making)	15	30.0
	Evaluating quality of care	15	30.0
**Areas for improvement** ^b^ **(multiple could apply)**			
	Functionality (eg, reports, graphs, and reminders)	13	26.0
	Integration with electronic medical records	9	18.0
	Patient and clinician engagement	8	16.0
	User-friendliness and accessibility	7	14.0
	None reported	14	28.0
**Clinical ePRO application not used anymore**		28	35.9
**Reasons why (multiple could apply)**			
	Lack of funding	12	42.9
	Logistical or organizational challenges	9	32.1
	Technical problems	8	28.6
	Change to different software for ePRO assessment	8	28.6
	Underutilization	3	10.7
	Lack of user-friendliness	2	7.1
	Change in clinical needs	1	3.6
**Requirements for reconsidering ePRO use^b^ (multiple could apply)**			
	Funding or resources	13	46.4
	Integration with existing clinical systems	5	17.9
	Improved usability	4	14.3
	Patient and clinician engagement	2	7.1
	None reported	9	32.1

^a^ePRO: electronic patient-reported outcome.

^b^These questions were a free text field the answers to which we categorized.

## Discussion

### Overview

In this study, we systematically reviewed studies reporting on ePRO assessment in clinical practice and present the results of a follow-up survey investigating the long-term use of the identified clinical ePRO applications. The results show a significant increase in publications on clinical ePRO applications over time including time trends towards app-based ePRO software, patient-centered care (data collection with feedback of PRO results to patients), and remote assessments. Information on the current use of identified ePRO applications could be gathered for about a third, of which 64% were reportedly still in use, primarily for monitoring, feedback of PRO results to patients, and research. Lack of funding was the main reason for discontinuation of ePRO application use, with more resources being the key requirement for reconsidering reuptake.

### ePRO Use in Clinical Practice and Time Trends

The review highlights the growing role of clinical ePRO applications in routine care, especially for monitoring symptoms and HRQOL during treatment and early aftercare. Unlike research-focused data collection, PRO monitoring and subsequent management by health care providers can offer direct patient benefits [8,9]. Increased ePRO publications over the last decade [28,29] were likely spurred by 2 landmark trials in 2017 showing survival benefits from remote ePRO monitoring [30,31]. Basch et al [30] demonstrated overall survival benefits in patients with solid tumors who were monitored via an ePRO application, where nurses received alerts when patients reported severe symptoms. Similarly, Denis et al [31] reported an improved overall survival of the ePRO arm when compared to standard of care. These results highlighted the potential of ePROs to improve outcomes, possibly driving their broader adoption. Clinical ePROs are increasingly used to provide patients with feedback on their scores, enabling them to actively manage their health and make informed care decisions [7,32].

Our follow-up survey found discrepancies between published reports of ePRO use and their actual use in clinical practice. This may be due to varied interpretations of survey categories, unreported use cases, or the evolving role of ePROs over time. For instance, quality of care evaluation was cited more in the survey (30%) than in studies (3%). Overall, our findings suggest that clinical ePRO use is broader and more diverse than reflected in current literature.

Our findings also show that website interfaces remain dominant; however, mobile apps are becoming increasingly more prevalent in the field of oncology, as Lu et al [33] showed in their review of 41 symptom-tracking apps. Apps can offer more accessible, user-friendly interfaces for patients and have shown notable growth in utilization. For example, a study by Pho et al [34] demonstrated a significant increase in mobile app log-ins over time, particularly in younger patient populations, leading to a higher overall frequency of ePRO use compared to users of the website interface. Looking ahead, one might expect patient apps to become even more prevalent and ePROs to become more often integrated into electronic health record systems to facilitate access for health care professionals.

### Sustainability of Clinical ePRO Applications

Participants in the follow-up survey reported that about two-thirds of the ePRO applications were still in use, with some applications being active for over 20 years. However, it is important to note that this represents only 23% of the total number of applications reviewed. Authors with ePRO applications still in active use may have been more likely to respond to our survey, potentially leading to an overestimation of sustainable use. Hence, the true rate of sustainable use likely falls between the 64% (50/78) of applications still in use according to our survey and the more conservative estimate of 23% (50/221) according to all applications initially identified. Additionally, other research shows that mobile apps in oncology are often no longer available once their respective research project is concluded [35].

Interestingly, some centers that discontinued their original ePRO applications reported to have transitioned to different software solutions for continued ePRO assessment. For example, in Denmark and Ontario, Canada, ePRO assessment was implemented on a national (regional) level showing the possibility of evolving and adapting ePRO applications [36,37].

Despite these encouraging examples, the sustainability of clinical ePRO applications remains a significant challenge, particularly due to financial constraints. The high initial costs of implementing clinical ePRO applications can often come without a clear direct return on investment, making it difficult to maintain these tools over the long term. Additionally, switching to another software or vendor can double these expenses, further straining budgets. The lack of follow-up financing was cited as the primary reason for discontinuing many applications. Some countries, such as France, are exploring reimbursement models for remote patient monitoring to offset these financial challenges that can also apply to ePRO applications in clinical care [38]. Notably, reimbursement has recently been granted for a clinical ePRO application in 33 cancer centers with an additional 18 about to follow, demonstrating the policy's potential impact on broader adoption [39].

In addition to financial barriers, integrating ePRO assessment into electronic health records and clinical workflows, the need for appropriate user support, and the demand for features like enhanced data visualization and real-time feedback to improve sustainability are frequently mentioned challenges [18,40]. Correspondingly, a review of 15 ePRO systems in nephrology identified sustained patient involvement, clinician champions and expanding existing electronic platforms to integrate ePROs as important factors for a sustainable ePRO implementation [41].

To address these challenges and promote long-term success, we recommend using established methodologies designed to support successful clinical implementation, where sustainability is a key outcome criterion (see, for example, [42-44]).

### Strength and Limitations

Our paper offers valuable insights into the long-term use and sustainability of clinical ePRO applications, backed by both a comprehensive literature review and follow-up survey data. Another key strength lies in the novel exploration of time trends over the past 20 years that illustrate the use of clinical ePRO applications in oncology. The key limitation of the follow-up survey and the sustainability analysis is potential response bias. Potentially, authors whose ePRO application is still in use might be more prone to reply to the survey, thereby biasing the analysis. We also identified an increased likelihood of a response from clinical ePRO application with a higher total number of published articles which can be seen as an indicator for a bias towards more active research groups. Hence, we have interpreted our sustainability analysis based on all the applications initially identified and provide a more conservative estimate based on the total number of identified applications. Furthermore, we found that specific regions were overrepresented among respondents therefore biasing our results geographically.

Inconsistent reporting of ePRO systems in the literature poses another challenge in comprehensively reporting on the current practices of ePRO applications in clinical care. The lack of a specific reporting framework (or limited compliance with more general guidelines like [45]) makes it difficult to compare studies and draw conclusions about best practices for ePRO implementation. However, ongoing efforts to develop specific guidelines for reporting patient-reported outcome measures in routine care offer hope for addressing this issue (Patient-Reported Outcome Measures—Guideline for Reporting In clinical Practice [46]).

### Conclusions

Our study presents data on the sustainability and growing use of clinical ePRO applications in routine cancer care, with the potential to improve symptom monitoring and patient-centered care. While many applications remain in use, financial challenges and integration barriers might limit widespread adoption. Addressing these obstacles through reimbursement models, improved workflow integration, and enhanced support features will be essential for the sustained success of clinical ePRO applications in oncology.

## Appendix

Multimedia Appendix 1 Additional methods and material.

Multimedia Appendix 2 Overview of the data extraction results (N=303).

Multimedia Appendix 3 Number of publications over time (annual overview).

## Abbreviations

EORTC: European Organisation for Research and Treatment of Cancer
ePRO: electronic patient-reported outcome
HRQOL: health-related quality of life
ISOQOL: International Society for Quality of Life Research
OR: odds ratio
PRISMA-ScR: Preferred Reporting Items for Systematic Reviews and Meta-Analyses extension for Scoping Reviews
PRO: patient-reported outcome
PRO-CTCAE: Patient-Reported Outcomes Version of the Common Terminology Criteria for Adverse Events

## Data Availability

All data from the review are available as supplementary material. Data from the follow-up survey are available in anonymized form upon reasonable request from the authors.
