# Supplementary material for: Sustainability and Time Trends in Electronic Patient-Reported Outcome Assessment in Routine Cancer Care: Systematic Scoping Review and Follow-Up Survey
Source: J Med Internet Res. 2025 Apr 25;27:e69398. doi: 10.2196/69398 (PMC12064961; doi:10.2196/69398)
Supplement: Multimedia Appendix 1 [file jmir_v27i1e69398_app1.pdf]

Supplementary Material

Table of Contents

Methods S1: Preferred Reporting Items for Systematic reviews and Meta-Analyses extension for Scoping Reviews (PRISMA-ScR) Checklist 2

Methods S2: Search Strategy 4

Methods S3: Follow-up Survey Case Report Form 5

Table S1: Location of cancer centres 6

Table S2: ePRO Software Tool Names 7

Table S3: Questionnaires used in the ePRO Software Tool 12

References: References included in the review 16

Methods S1: Preferred Reporting Items for Systematic reviews and Meta-Analyses extension for Scoping Reviews (PRISMA-ScR) Checklist

SECTION	ITEM	PRISMA-ScR CHECKLIST ITEM	REPORTED ON PAGE #
**TITLE**			
Title	1	Identify the report as a scoping review.	1
**ABSTRACT**			
Structured summary	2	Provide a structured summary that includes (as applicable): background, objectives, eligibility criteria, sources of evidence, charting methods, results, and conclusions that relate to the review questions and objectives.	1-2
**INTRODUCTION**			
Rationale	3	Describe the rationale for the review in the context of what is already known. Explain why the review questions/objectives lend themselves to a scoping review approach.	2
Objectives	4	Provide an explicit statement of the questions and objectives being addressed with reference to their key elements (e.g., population or participants, concepts, and context) or other relevant key elements used to conceptualize the review questions and/or objectives.	2
**METHODS**			
Protocol and registration	5	Indicate whether a review protocol exists; state if and where it can be accessed (e.g., a Web address); and if available, provide registration information, including the registration number.	N/A
Eligibility criteria	6	Specify characteristics of the sources of evidence used as eligibility criteria (e.g., years considered, language, and publication status), and provide a rationale.	2
Information sources*	7	Describe all information sources in the search (e.g., databases with dates of coverage and contact with authors to identify additional sources), as well as the date the most recent search was executed.	2
Search	8	Present the full electronic search strategy for at least 1 database, including any limits used, such that it could be repeated.	Multimedia Appendix Methods S2
Selection of sources of evidence†	9	State the process for selecting sources of evidence (i.e., screening and eligibility) included in the scoping review.	2
Data charting process‡	10	Describe the methods of charting data from the included sources of evidence (e.g., calibrated forms or forms that have been tested by the team before their use, and whether data charting was done independently or in duplicate) and any processes for obtaining and confirming data from investigators.	2-3
Data items	11	List and define all variables for which data were sought and any assumptions and simplifications made.	2-3
Critical appraisal of individual sources of evidence§	12	If done, provide a rationale for conducting a critical appraisal of included sources of evidence; describe the methods used and how this information was used in any data synthesis (if appropriate).	N/A
Synthesis of results	13	Describe the methods of handling and summarizing the data that were charted.	3
**RESULTS**			

SECTION	ITEM	PRISMA-ScR CHECKLIST ITEM	REPORTED ON PAGE #
Selection of sources of evidence	14	Give numbers of sources of evidence screened, assessed for eligibility, and included in the review, with reasons for exclusions at each stage, ideally using a flow diagram.	3-4
Characteristics of sources of evidence	15	For each source of evidence, present characteristics for which data were charted and provide the citations.	Multimedia Appendix 2
Critical appraisal within sources of evidence	16	If done, present data on critical appraisal of included sources of evidence (see item 12).	N/A
Results of individual sources of evidence	17	For each included source of evidence, present the relevant data that were charted that relate to the review questions and objectives.	Multimedia Appendix 2
Synthesis of results	18	Summarize and/or present the charting results as they relate to the review questions and objectives.	3-10
**DISCUSSION**			
Summary of evidence	19	Summarize the main results (including an overview of concepts, themes, and types of evidence available), link to the review questions and objectives, and consider the relevance to key groups.	10-11
Limitations	20	Discuss the limitations of the scoping review process.	11
Conclusions	21	Provide a general interpretation of the results with respect to the review questions and objectives, as well as potential implications and/or next steps.	11
**FUNDING**			
Funding	22	Describe sources of funding for the included sources of evidence, as well as sources of funding for the scoping review. Describe the role of the funders of the scoping review.	11

JBI = Joanna Briggs Institute; PRISMA-ScR = Preferred Reporting Items for Systematic reviews and Meta-Analyses extension for Scoping Reviews.

* Where *sources of evidence* (see second footnote) are compiled from, such as bibliographic databases, social media platforms, and Web sites.

† A more inclusive/heterogeneous term used to account for the different types of evidence or data sources (e.g., quantitative and/or qualitative research, expert opinion, and policy documents) that may be eligible in a scoping review as opposed to only studies. This is not to be confused with *information sources* (see first footnote).

‡ The frameworks by Arksey and O'Malley (6) and Levac and colleagues (7) and the JBI guidance (4, 5) refer to the process of data extraction in a scoping review as data charting.

§ The process of systematically examining research evidence to assess its validity, results, and relevance before using it to inform a decision. This term is used for items 12 and 19 instead of "risk of bias" (which is more applicable to systematic reviews of interventions) to include and acknowledge the various sources of evidence that may be used in a scoping review (e.g., quantitative and/or qualitative research, expert opinion, and policy document).

From: Tricco AC, Lillie E, Zarin W, O'Brien KK, Colquhoun H, Levac D, et al. PRISMA Extension for Scoping Reviews (PRISMAScR): Checklist and Explanation. Ann Intern Med. 2018;169:467–473. doi: 10.7326/M18-0850.

Methods S2: Search Strategy

Web of Science

Clinical routine:
(TI=("clinical care" OR "patient care" OR "health care" OR "clinical routine" OR "routine care")) OR AB=("clinical care" OR "patient care" OR "health care" OR "clinical routine" OR "routine care")

ePRO:
(TI=(electronic OR electronical OR web OR webbased OR web-based OR "web based" OR multimedia OR multimedial OR internet OR computer OR online OR distance OR mobile OR phone OR smartphone OR cellphone OR digital OR handheld OR tablet OR interactive OR ePRO or e-PRO OR remote)) OR AB=(electronic OR electronical OR web OR webbased OR web-based OR "web based" OR multimedia OR multimedial OR internet OR computer OR online OR distance OR mobile OR phone OR smartphone OR cellphone OR digital OR handheld OR tablet OR interactive OR ePRO or e-PRO OR remote)

PRO/QOL
(TI=("patient-reported" OR "patient reported" OR PRO OR "self-reported" OR "self reported" OR "quality of life" OR QOL OR HRQOL OR HRQL OR symptom OR symptoms)) OR (AB=("patient-reported" OR "patient reported" OR PRO OR "self-reported" OR "self reported" OR "quality of life" OR QOL OR HRQOL OR HRQL OR symptom OR symptoms)

Cancer:
(TI=(oncolog* OR neoplas* OR cancer*)) OR AB=(oncolog* OR neoplasm* OR cancer*)

PubMed:

("quality of life"[Title/Abstract] OR "health related quality of life"[Title/Abstract] OR "life quality"[Title/Abstract] OR "health outcomes"[Title/Abstract] OR "health status"[Title/Abstract] OR "patient reported symptom"[Title/Abstract] OR "patient reported outcomes"[Title/Abstract] OR "patient reported outcome"[Title/Abstract] OR "PRO"[Title/Abstract] OR "PROs"[Title/Abstract] OR "HRQL"[Title/Abstract] OR "QOL"[Title/Abstract] OR "HRQOL"[Title/Abstract] OR "self report"[Title/Abstract] OR "self assessment"[Title/Abstract] OR "self disclosure"[Title/Abstract])

AND ("clinical care"[Title/Abstract] OR "patient care"[Title/Abstract] OR "health care"[Title/Abstract] OR "clinical routine"[Title/Abstract] OR "routine care"[Title/Abstract] OR "clinical practice"[Title/Abstract] OR "clinical cancer care"[Title/Abstract] OR "daily practice"[Title/Abstract] OR "routine monitoring"[All Fields])

AND ("oncology"[Title/Abstract] OR "cancer"[Title/Abstract] OR "tumor"[Title/Abstract] OR "neoplasm"[Title/Abstract])

AND ("electronic"[Title/Abstract] OR "ePRO"[Title/Abstract] OR "e-PRO"[Title/Abstract] OR "electronical"[Title/Abstract] OR "web"[Title/Abstract] OR "webbased"[Title/Abstract] OR "multimedia"[Title/Abstract] OR "multimedial"[Title/Abstract] OR "internet"[Title/Abstract] OR "computer"[Title/Abstract] OR "online"[Title/Abstract] OR "distance"[Title/Abstract] OR "mobile"[Title/Abstract] OR "phone"[Title/Abstract] OR "smartphone"[Title/Abstract] OR "cellphone"[Title/Abstract] OR "digital"[Title/Abstract] OR "handheld"[Title/Abstract] OR "tablet"[Title/Abstract] OR "interactive"[Title/Abstract] OR "remote"[Title/Abstract])

Methods S3: Follow-up Survey Case Report Form

1. Has the ePRO assessment/system only been used in the context of the published study or also outside of the original study or in other contexts?

( ) only used in the published study
( ) used in the published study and in further applications/contexts

2. Is the ePRO assessment/system we have referenced in our email still in use? (Select one answer)

( ) Yes
( ) No

IF NO, please only answer the questions 3 – 4

3. Why do you no longer use the ePRO assessment/system? (Multiple answers possible)

( ) Technical or IT problems (e.g., problems with the ePRO system)
( ) Lack of user-friendliness of the system
( ) Change in clinical needs
( ) Lack of funding
( ) Lack of use of the tool in clinical practice
( ) Logistical challenges (e.g., no dedicated personnel or resources)
( ) Other (please specify): ___________

4. What would be necessary for you to consider using ePRO assessments or an ePRO system again? (e.g., would require dedicated funding, administrative support, more patient engagement, more user-friendly software, ...)

Please specify: ___________

IF YES, please only answer the questions 5 – 9

5. For how long have you been using the ePRO assessment/system?

Please specify: ___________ year(s)

6. What are the questionnaires (patient-reported outcome measures, PROMs) assessed in your ePRO assessment/system used for? (Multiple answers possible)

( ) Clinical Routine: Screening Tools (One-time PRO assessment with feedback to clinician)
( ) Clinical Routine: Monitoring Tools (data collection over time with feedback to clinician)
( ) Clinical Routine: Patient-centered care (data collection with feedback to patients and clinicians)
( ) Clinical Routine: Decision aids (e.g., decision aid that presents PRO information to patients)
( ) Clinical Routine: Facilitating multidisciplinary team communications
( ) Evaluating quality of care (Pooled data from patients from within the practice)
( ) Research or clinical studies
( ) Other (please specify): ___________

7. On average, how many patients participate in the ePRO assessments each month? (i.e., the number of patients that complete a questionnaire or use the system) (Select one answer)

( ) 0 to 50 patients
( ) 51 to 200 patients
( ) 201 to 500 patients
( ) More than 500 patients

8. How satisfied are you with the ePRO assessments/system? (Select one answer)

( ) Not at all
( ) A little bit
( ) Quite a bit
( ) Very much

9. If you could improve some aspects of the ePRO assessments or the software, what would you improve?

Please specify: ___________

Table S1: Location of cancer centres

Country	Number of occurrences
USA	104
United Kingdom	26
The Netherlands	23
Canada	16
Germany	15
Sweden	13
Australia	12
Austria	11
Italy	9
Denmark	7
Multiple countries	9
France	6
Norway	6
Republic of Korea	6
Finland	5
Belgium	4
Iran	4
Switzerland	4
China	3
Japan	3
not reported	3
Taiwan	3
Brazil	2
Singapore	2
Spain	2
Chile, Iceland, Portugal, Thailand, Uruguay	1 (each)

Table S2: ePRO Software Tool Names

**Tool name**	***N* of publications**
CHES	16
Interaktor	10
Patient Care Monitor	8
PROMPT-Care	7
Advanced Symptom Management System (ASyMS)	6
Epic MyChart	6
Kaiku	6
Carevive	5
eRAPID	5
Noona	5
OncoKompas	4
REDCap	4
STAR	4
KLIK	3
OncoLink	3
PatientViewpoint	3
PROFILES	3
Sisom	3
AmbuFlex	2
AMTRA	2
Cancer self-management system (CanSelfMan)	2
CAPRI	2
consilium care app	2
EirV3	2
Electronic Self-report Assessment - Cancer (ESRA-C)	2
Electronic symptom management program (eSyM)	2
Epic	2
HN-STAR	2
imPROVE	2
MSK Engage	2
MyChristie-MyHealth	2
OncoQuest	2

PACE (TM)	2
PediQUEST	2
Pit-a-Pat	2
SAM	2
SPARK	2
Symptom Assessment and Management Intervention (SAMI-L) System	2
Transmural Oncologal Support (TOS)	2
+Contigo	1
3H-guardian	1
Handheld computer system (HCS)	1
ACESO	1
AMOR Mama	1
BD4QoL	1
Be-with-You	1
Breast cancer patient support system (BPSS)	1
Breast cancer self-management support (BCSMS)	1
bridges	1
Cancer Web Portal	1
CANKADO	1
Capturing and Analyzing Sensor and Self-Report Data for Clinicians and Researchers (COMPASS)	1
CEASE	1
ChemOtheRapy Assistant (CORA)	1
Choice ITPA	1
Circadian Questions (CQ)	1
Clinet	1
CMyLife	1
Comfort	1
COMPASS-CP CFC	1
Composite Holistic Needs Assessment Adaptive Tool-Prostate (CHAT-P)	1
Computerized Symptom Capture Tool (C-SCAT)	1
Connect	1
CORA	1
Cureety	1

DAPROCAdata	1
Digital BioMarkers for Clinical Impact (DigiBioMarC™)	1
Digital Supportive Care Awareness and Navigation (D-SCAN)	1
Distress Assessment and Response Tool (DART)	1
Docoboweb	1
elcss-ql	1
Electronic Patient Questionnaire (EPQ)	1
E-MOSAIC palm	1
Engage	1
e-OncoSalud	1
EPE-LE	1
ePRO-CTCAE app	1
ePROhub	1
Gemstracker	1
Ghasedak	1
GIMEMA-ALLIANCE platform	1
Guardian Angel	1
Handheld Symptom Management (HSM)	1
Healthcare Monitor	1
HealthWeaver Mobile	1
HeNeA	1
HOPE	1
ICOnnecta't	1
IMPROVED	1
Improving Patient Experience and Health Outcome Collaborative (iPEHOC)	1
InSight Care	1
INSPIRE	1
Interactive Symptom Assessment and Collection (ISAAC)	1
Kræftværket	1
LivingWith	1
Lung Cancer Care	1
mBraze	1
MoodUP	1
My University Clinic (MUC)	1

NeuroDetect 1.0	1
NOOM, Walkon, Efilcare	1
Onco-TreC	1
Oral care mobile app	1
Outcome Registry Intervention and Operation Network (ORION)	1
Outcomes4Me	1
OWise	1
PainCheck	1
PainRELife	1
Patient Assessment, Care & Education (PACE) System™	1
Patient Voices	1
PatientConcept	1
PaTOS	1
PiiA	1
Polaris Oncology Survivorship Transition (POST) System	1
Postoperative symptom interventiontool (POSIT)	1
PRISMS	1
PRO-CTCAE Administration System (mPROS)	1
PROMetheus	1
PROQOL	1
Prospective Outcomes and Support Initiative (POSI)	1
PROSS	1
PRO-TECT	1
Putting Patients First (PPF)	1
QoL-Profiler	1
QTool	1
RESPONSE System	1
RetinoQuest	1
RT-CAMSS	1
ScreenIT	1
SIS.NET	1
Smart After-Care	1
SmartSurvivor	1
Strength Through Insight	1

Symptom Care and Management System (SCMS)	1
Symptom Care at Home (SCH)	1
Text4Hope-Cancer Care	1
The ACCESS system	1
THRIVE	1
Tonic	1
UNC PRO Core system	1
Upper Digestive Disease Application (UDD App)	1
VPROMS	1
WalkON	1
WebChoice	1
WHOMS	1
not reported	55

Table S3: Questionnaires used in the ePRO Software Tool

Questionnaires	*N* of publications
EORTC questionnaires (C30 and/or modules)	77
PRO-CTCAE	65
EQ-5D	28
Own development	27
FACIT questionnaires	26
ESAS	23
Distress Thermometer (& Problem List)	22
Hospital Anxiety and Depression Scale (HADS)	19
PROMIS questionnaires [a]	15
Patient Health Questionnaire [a]	14
Memorial Symptom Assessment Scale[a]	11
MD Anderson Symptom Inventory (MDASI)[a]	10
Pediatric Quality of Life Inventory (PedsQL)[a]	8
Expanded Prostate Cancer Index Composite (EPIC)[a]	7
Shorth Form Health Survey[a]	7
Patient Care Monitor[a]	6
Supportive Care Needs Survey	6
Visual Analogue Scale[a]	6
BREAST-Q	5
Numerical Rating Scale[a]	5
Brief Pain Inventory (BPI)	4
Insomnia Severity Index (ISI)	4
Generalized Anxiety Disorder (GAD-7)[a]	3
Patient Generated Subjective Global Assessment (PG-SGA)	3
Social Difficulties Inventory (SDI-21)	3
Symptom Distress Scale (SDS)	3
Bladder Cancer Index (BCI)	2
Brief Illness Perception Questionnaire (B-IPQ)	2
Canadian Problem Checklist (CPC)	2
Diverticulitis Quality of Life (DV-QOL)	2
e-Diary	2
Hornheider Screening Instrument (HSI)	2
Patient Activation Measure (PAM-13)	2

Quality of Life in Adult Cancer Survivors (QLACS)	2
Rotterdam Symptom Checklist – Activity Subscale (RSC-Activity)	2
Self-Efficacy for Managing Chronic Disease	2
Self-Efficacy Scale	2
Strengths and Difficulties Questionnaire (SDQ)	2
Symptom Screening in Pediatrics Tool (SSPedi)	2
15D	1
Adherence to Refills and Medications Scale (ARMS-7)	1
Adult Global Health 10 survey (Global-10 v.1.0/1.1)	1
AET medication Adherence (adapted from MAQ)	1
Arthritis Self-Efficacy Scale	1
ASyMSB-YG PDA (personal digital assistant) questionnaire	1
Beck's Depression Inventory (BDI)	1
Breast Cancer and Lymphedema Symptom Index (BCLE-SEI)	1
Brief Fatigue Inventory (BFI)	1
Cancer Behavior Inventory - Brief (CBI-B)	1
Cancer Fatigue Scale (CFS)	1
Cancer survivor identity question	1
Cancer Survivor Profile for breast cancer survivors (CSPro-BC)	1
Cancer-related Dysfunctional Beliefs about Sleep	1
CARERQOL-7D	1
CCCQ	1
CEASE tool	1
Chemotherapy Symptom Assessment Scale	1
Chemotherapy Toxicity Self-Assessment Questionnaire	1
Colorectal cancer module	1
Communicative and Critical Health LiteracyScale (CCHL)	1
Constipation severity measure	1
Constructs Empowering Outcomes questionnaire	1
Control Preference Scale	1
CTX Toxicity Self-Assessment Questionnaire (CTAQ)	1
DASH	1
Demands of BMT Inventory	1
Disability Index [HAQ-DI]	1

ECOG	1
Edmonton Classification System for Cancer Pain (ECS–CP)	1
FACES pain scale	1
Fear of Progression Questionnaire-Short Form	1
Fertility Concerns Questionnaire	1
Functional Health Literacy Scale (FHL)	1
Functional Oral Intake Scale (FOIS)	1
General Self-Efficacy scale	1
General, Physical Well-Being score	1
GHQ-28	1
Global-10	1
Health Education Impact Questionnaire (heiQ)	1
Health Perceptions Questionnaire	1
Health utility score (HUS)	1
HRQL-PSY	1
HSCT-specific symptom inventory	1
Illness Perception Questionnaire-Revised (IPQ-R)	1
Individual Care scale	1
International Physical Activity Questionnaire (IPAQ)	1
International Prostate Symptom Score (IPSS)	1
IPAQ-SF	1
Karnofsky index	1
LASA	1
Lung Cancer Symptom Scale	1
MAC	1
MARS-1	1
Mental adjustment to cancer scale	1
Mini Nutritional Assessment (MNA)	1
Multidimensional Fatigue Inventory (MFI)	1
NSQ	1
Oxaliplatin-Associated Neurotoxicity Questionnaire (OANQ)	1
Pain and Discomfort	1
Pain Catastrophizing Scale	1
Pain Self-Efficacy Questionnaire (PSEQ)	1

Pain-Therapy Impact Questionnaire (pain-TIQ scale)	1
Patient Assessment of Chronic Illness Care questionnaire (PACIC)	1
Patient Concern Inventory	1
Patient Satisfaction Questionnaire (PSQ-18)	1
Patient-Reported Symptom Monitoring (PRSM)	1
Pearlin & Schooler Mastery Scale	1
Pediatric Distress Thermometer	1
Perceived Efficacy Patient-Physician Interactions scale	1
Personal Health Questionnaire Depression Scale (PHQ-8)	1
Physical performance (ECOG)	1
Polaris Oncology Survivorship Transition Patient Assessment	1
PRO - Lung symptom questionnaire	1
Profile of Mood States	1
PRO-Onc	1
Quality of Recovery (QoR-9)	1
Sense of Coherence Scale	1
Short Inflammatory Bowel Disease Questionnaire (SIBDQ)	1
Sisom	1
SOC	1
SPARC	1
Standard depression and anxiety screening tools	1
State-Trait Anxiety Inventory Form Y	1
SUPPH	1
Symptom Checklist-90-R	1
The Cancer and Treatment Distress scale	1
The Ferrans and Powers Quality of Life Index Cancer Version III (QLI)	1
UDD questionnaire	1
UKONS 24-hour triage tool	1
UWQOLv4	1
Ways of Coping (WAYS)	1
World Health Organization Disability Assessment Schedule (WHODAS)	1
World Health Organization oral toxicity scale	1
World Health Organization Well-Being Index (WHO-5)	1

[a] all versions

References: References included in the review

1 A Payne, A Horne, N Bayman, *et al.* Patient and clinician-reported experiences of using electronic patient reported outcome measures (ePROMs) as part of routine cancer care. *Journal of patient-reported outcomes* 2023; **7**: 42.

2 Abdelmutti N, Brual J, Papadakos J, *et al.* Implementation of a comprehensive smoking cessation program in cancer care. *Current oncology (Toronto, Ont)* 2019; **26**: 361–8.

3 Abernethy AP, Herndon JE, Wheeler JL, *et al.* Improving Health Care Efficiency and Quality Using Tablet Personal Computers to Collect Research-Quality, Patient-Reported Data. *HEALTH SERVICES RESEARCH* 2008; **43**: 1975–91.

4 Abernethy AP, Herndon JE, Wheeler JL, *et al.* Feasibility and Acceptability to Patients of a Longitudinal System for Evaluating Cancer-Related Symptoms and Quality of Life: Pilot Study of an e/Tablet Data-Collection System in Academic Oncology. *Journal of pain and symptom management* 2009; **37**: 1027–38.

5 Absolom K, Holch P, Warrington L, *et al.* Electronic patient self-Reporting of Adverse-events: Patient Information and aDvice (eRAPID): a randomised controlled trial in systemic cancer treatment. *BMC cancer* 2017; **17**. DOI:10.1186/s12885-017-3303-8.

6 Adeola B-A, Ivana D, Orlando R, *et al.* Supporting cancer patients to self-manage: Extent of use and perceptions of 'trusted' online self-management resources. *Patient education and counseling* 2022; **105**: 2240–7.

7 Agboola S, Flanagan C, Searl M, Elfiky A, Kvedar J, Jethwani K. Improving outcomes in cancer patients on oral anti-cancer medications using a novel mobile phone-based intervention: study design of a randomized controlled trial. *JMIR research protocols* 2014; **3**: e79.

8 Agyapong VIO, Hrabok M, Shalaby R, *et al.* Closing the COVID-19 Psychological Treatment Gap for Cancer Patients in Alberta: Protocol for the Implementation and Evaluation of Text4Hope-Cancer Care. *JMIR research protocols* 2020; **9**. DOI:10.2196/20240.

9 Albaba H, Barnes TA, Veitch Z, *et al.* Acceptability of Routine Evaluations Using Patient-Reported Outcomes of Common Terminology Criteria for Adverse Events and Other Patient-Reported Symptom Outcome Tools in Cancer Outpatients: Princess Margaret Cancer Centre Experience. *The oncologist* 2019; **24**: e1219–27.

10 Appleton R, Nanton V, Ahmed N, *et al.* A Web-Based Prostate Cancer-Specific Holistic Needs Assessment (CHAT-P): Multimethod Study From Concept to Clinical Practice. *JMIR CANCER*; **8**. DOI:10.2196/32153.

11 Appleyard SE, Larkin MJW, Stewart EM, Minton O, Gilbert DC. Digital Medicine in Men with Advanced Prostate Cancer- A Feasibility Study of Electronic Patient-reported Outcomes in Patients on Systemic Treatment. *Clinical oncology (Royal College of Radiologists (Great Britain))* 2021; **33**: 751–60.

12 Armstrong TS, Vera-Bolanos E, Acquaye A, Gilbert MR, Mendoza TR. Impact of recall period on primary brain tumor patient's self-report of symptoms. *Neuro-oncology practice* 2014; **1**: 55–63.

13 Arriola E, Jaal J, Edvardsen A, *et al.* Feasibility and User Experience of Digital Patient Monitoring for Real-World Patients With Lung or Breast Cancer. *The oncologist* 2023. DOI:10.1093/oncolo/oyad289.

14 Arthur J, Yennurajalingam S, Nguyen L, *et al.* The routine use of the Edmonton Classification System for Cancer Pain in an outpatient supportive care center. *Palliative & supportive care* 2015; **13**: 1185–92.

15 Arts LPJ, van de Poll-Franse LV, van den Berg SW, *et al.* Lymphoma InterVEntion (LIVE) - patient-reported outcome feedback and a web-based self-management intervention for patients with lymphoma: study protocol for a randomised controlled trial. *Trials* 2017; **18**. DOI:10.1186/s13063-017-1943-2.

16 Ashley L, Jones H, Forman D, *et al.* Feasibility test of a UK-scalable electronic system for regular collection of patient-reported outcome measures and linkage with clinical cancer registry data: the electronic

Patient-reported Outcomes from Cancer Survivors (ePOCS) system. *BMC medical informatics and decision making* 2011; **11**: 1–10.

17 Baggott C, Baird J, Hinds P, Ruland CM, Miaskowski C. Evaluation of Sisom: A computer-based animated tool to elicit symptoms and psychosocial concerns from children with cancer. *European journal of oncology nursing : the official journal of European Oncology Nursing Society* 2015; **19**: 359–69.

18 Bamgboje-Ayodele A, Avery S, Pearson J, *et al.* Adapting an integrated care pathway for implementing electronic patient reported outcomes assessment in routine oncology care: Lessons learned from a case study. *JOURNAL OF EVALUATION IN CLINICAL PRACTICE* 2022; **28**: 1072–83.

19 Barlund M, Takala L, Tianen L, Kellokumpu-Lehtinen PL. Real-world Evidence of Implementing eHealth Enables Fluent Symptom-based Follow-up of a Growing Number of Patients with Breast Cancer with the Same Healthcare Resources. *Clinical breast cancer* 2022; **22**: 261–8.

20 Basch E, Artz D, Dulko D, *et al.* Patient online self-reporting of toxicity symptoms during chemotherapy. *Journal of clinical oncology : official journal of the American Society of Clinical Oncology* 2005; **23**: 3552–61.

21 Basch E, Stover AM, Schrag D, *et al.* Clinical Utility and User Perceptions of a Digital System for Electronic Patient-Reported Symptom Monitoring During Routine Cancer Care: Findings From the PRO-TECT Trial. *JCO clinical cancer informatics* 2020; **4**: 947–57.

22 Baseman J, Revere D, Baldwin L-M. A Mobile Breast Cancer Survivorship Care App: Pilot Study. *JMIR cancer* 2017; **3**: e14.

23 Belarmino A, Walsh R, Alshak M, Patel N, Wu R, Hu JC. Feasibility of a Mobile Health Application To Monitor Recovery and Patient-reported Outcomes after Robot-assisted Radical Prostatectomy. *European urology oncology* 2019; **2**: 425–8.

24 Bennett MI, Allsop MJ, Allen P, *et al.* Pain self-management interventions for community-based patients with advanced cancer: a research programme including the IMPACCT RCT. *Programme Grants for Applied Research* 2021; published online Dec. DOI:10.3310/pgfar09150.

25 Bergqvist J, Lundstrom S, Wengstrom Y. Patient interactive digital support for women with adjuvant endocrine therapy in order to increase compliance and quality of life. *Supportive care in cancer : official journal of the Multinational Association of Supportive Care in Cancer* 2021; **29**: 491–7.

26 Berry DL, Hong F, Halpenny B, *et al.* Electronic self-report assessment for cancer and self-care support: results of a multicenter randomized trial. *Journal of clinical oncology* 2014; **32**: 199.

27 Bielli E, Carminati F, La Capra S, Lina M, Brunelli C, Tamburini M. A Wireless Health Outcomes Monitoring System (WHOMS): development and field testing with cancer patients using mobile phones. *BMC medical informatics and decision making* 2004; **4**: 7.

28 Billingy NE, Tromp V, Veldhuijzen E, *et al.* SYMptom monitoring with Patient-Reported Outcomes using a web application among patients with Lung cancer in the Netherlands (SYMPRO-Lung): study protocol for a stepped-wedge randomised controlled trial. *BMJ open* 2021; **11**. DOI:10.1136/bmjopen-2021-052494.

29 Blum D, Koeberle D, Ribi K, *et al.* Electronic monitoring of symptoms and syndromes associated with cancer: methods of a randomized controlled trial SAKK 95/06 E-MOSAIC. *BMC palliative care* 2012; **11**: 19.

30 Bouma G, Hosson LD, van Woerkom CE, *et al.* Web-based information and support for patients with a newly diagnosed neuroendocrine tumor: a feasibility study. *Supportive care in cancer : official journal of the Multinational Association of Supportive Care in Cancer* 2017; **25**: 2075–83.

31 Bradford N, Condon P, Pitt E, Tyack Z, Alexander K. Optimising symptom management in children with cancer using a novel mobile phone application: protocol for a controlled hybrid effectiveness implementation trial (RESPONSE). *BMC health services research* 2021; **21**. DOI:10.1186/s12913-021-06943-x.

32 Breen S, Ritchie D, Schofield P, *et al.* The Patient Remote Intervention and Symptom Management System (PRISMS) - a Telehealth-mediated intervention enabling real-time monitoring of chemotherapy side-effects in patients with haematological malignancies: study protocol for a randomised controlled trial. *Trials* 2015; **16**. DOI:10.1186/s13063-015-0970-0.

33 Bronserud MM, Iachina M, Green A, Groenvold M, Dorflinger L, Jakobsen E. Patient-reported outcomes (PROs) in lung cancer: Experiences from a nationwide feasibility study. *Lung cancer (Amsterdam, Netherlands)* 2019; **128**: 67–73.

34 Brunelli C, Borreani C, Caraceni A, *et al.* PATIENT VOICES, a project for the integration of the systematic assessment of patient reported outcomes and experiences within a comprehensive cancer center: a protocol for a mixed method feasibility study. *Health and quality of life outcomes* 2020; **18**. DOI:10.1186/s12955-020-01501-1.

35 Bulls HW, Chang P-H, Brownstein NC, *et al.* Patient-reported symptom burden in routine oncology care: Examining racial and ethnic disparities. *Cancer reports (Hoboken, NJ)* 2022; **5**: e1478.

36 Bult MK, van Bindsbergen KLA, Schepers SA, *et al.* Health-Related Quality of Life of Adolescents with Cancer During the First Year of Treatment. *Journal of adolescent and young adult oncology* 2019; **8**: 616–22.

37 Bush N, Donaldson G, Moinpour C, *et al.* Development, feasibility and compliance of a web-based system for very frequent QOL and symptom home self-assessment after hematopoietic stem cell transplantation. *Quality of life research : an international journal of quality of life aspects of treatment, care and rehabilitation* 2005; **14**: 77–93.

38 Carhuapoma LR, Thayer WM, Elmore CE, *et al.* Employing a mobile health decision aid to improve decision-making for patients with advanced prostate cancer and their decision partners/proxies: the CHAMPION randomized controlled trial study design. *Trials* 2021; **22**. DOI:10.1186/s13063-021-05602-0.

39 Castillo C, Alfonso A, Dapueto J, Camejo N, Silva M. Inclusion of information technology-based assessments of health-related quality of life in routine oncology practice in Uruguay. *JOURNAL OF PATIENT-REPORTED OUTCOMES*; **6**. DOI:10.1186/s41687-022-00458-7.

40 Caston NE, Franks JA, Balas N, *et al.* Evaluating Nurses' Time to Response by Severity and Cancer Stage in a Remote Symptom Monitoring Program for Patients With Breast Cancer. *JCO clinical cancer informatics* 2023; **7**: e2300015.

41 Cavalieri S, Vener C, LeBlanc M, *et al.* A multicenter randomized trial for quality of life evaluation by non-invasive intelligent tools during post-curative treatment follow-up for head and neck cancer: Clinical study protocol. *Frontiers in oncology* 2023; **13**: 1048593.

42 Chaar M, Yost K, Lee M, *et al.* Developing & integrating a mobile application tool into a survivorship clinic for esophageal cancer patients. *JOURNAL OF THORACIC DISEASE*; **15**: 2240-+.

43 Chan M, Fai A, Emily D, Minh Cam C. An online S ymptom C are and M anagement S ystem to monitor and support patients receiving chemotherapy: A pilot study. *INTERNATIONAL JOURNAL OF NURSING PRACTICE* 2013; **19**: 14–8.

44 Chen CS, Kim J, Garg N, *et al.* Chemotherapy-Induced Peripheral Neuropathy Detection via a Smartphone App: Cross-sectional Pilot Study. *JMIR mHealth and uHealth* 2021; **9**. DOI:10.2196/27502.

45 Cheng ASK, Liu X, Ng PHF, Kwok CTT, Zeng Y, Feuerstein M. Breast cancer application protocol: a randomised controlled trial to evaluate a self-management app for breast cancer survivors. *BMJ open* 2020; **10**: e034655.

46 Cleeland C, Moos R, Walker MS, *et al.* Burden of symptoms associated with development of metastatic bone disease in patients with breast cancer. *Supportive care in cancer : official journal of the Multinational Association of Supportive Care in Cancer* 2016; **24**: 3557–65.

47 Cnossen IC, Bree R, Rinkel RNPM, *et al.* Computerized monitoring of patient-reported speech and swallowing problems in head and neck cancer patients in clinical practice. *Supportive care in cancer : official journal of the Multinational Association of Supportive Care in Cancer* 2012; **20**: 2925–31.

48 Collado-Borrell R, Escudero-Vilaplana V, Ribed A, *et al.* Effect of a Mobile App for the Pharmacotherapeutic Follow-Up of Patients With Cancer on Their Health Outcomes: Quasi-Experimental Study. *JMIR mHealth and uHealth* 2020; **8**. DOI:10.2196/20480.

49 Cook S, Vettese E, Tomlinson GA, *et al.* Feasibility of a randomized controlled trial of symptom screening and feedback to healthcare providers compared with standard of care using the SPARK platform. *Supportive care in cancer : official journal of the Multinational Association of Supportive Care in Cancer* 2020; **28**: 2729–34.

50 Cook S, Vettese E, Soman D, *et al.* Initial development of Supportive care Assessment, Prioritization and Recommendations for Kids (SPARK), a symptom screening and management application. *BMC medical informatics and decision making* 2019; **19**: 9.

51 Cooley ME, Blonquist TM, Catalano PJ, *et al.* Feasibility of using algorithm-based clinical decision support for symptom assessment and management in lung cancer. *Journal of pain and symptom management* 2015; **49**: 13–26.

52 Cowan RA, Suidan RS, Andikyan V, *et al.* Electronic patient-reported outcomes from home in patients recovering from major gynecologic cancer surgery: A prospective study measuring symptoms and health-related quality of life. *Gynecologic oncology* 2016; **143**: 362–6.

53 Crafoord MT, Fjell M, Sundberg K, Nilsson M, Langius-Eklof A. Engagement in an Interactive App for Symptom Self-Management during Treatment in Patients With Breast or Prostate Cancer: Mixed Methods Study. *Journal of medical Internet research* 2020; **22**. DOI:10.2196/17058.

54 Crockett C, Price J, Pham M, *et al.* Experience With the Routine Use of Electronic Patient-Reported Outcome Measures for Patients With Lung Cancer. *JCO clinical cancer informatics* 2023; **7**: e2200150.

55 Da Cruz F, Faria ET, Ghobad PC, Alves LYM, Dos Reis PED. A Mobile App (AMOR Mama) for Women With Breast Cancer Undergoing Radiation Therapy: Functionality and Usability Study. *Journal of medical Internet research* 2021; **23**. DOI:10.2196/24865.

56 Daly B, Kuperman G, Zervoudakis A, *et al.* InSight Care Pilot Program: Redefining Seeing a Patient. *JCO oncology practice* 2020; **16**: 675-+.

57 Della Mea V, Momi I, Aprile G, *et al.* Feasibility study of a web application for self-report of anticancer treatment toxicities. In: Medical Informatics in a United and Healthy Europe. IOS Press, 2009: 562–6.

58 Demedts I, Himpe U, Bossuyt J, *et al.* Clinical implementation of value based healthcare: Impact on outcomes for lung cancer patients. *Lung cancer (Amsterdam, Netherlands)* 2021; **162**: 90–5.

59 Denis F, Basch E, Septans A-L, *et al.* Two-year survival comparing web-based symptom monitoring vs routine surveillance following treatment for lung cancer. *Jama* 2019; **321**: 306–7.

60 Dronkers EAC, Jong RJB, van der Poel EF, Sewnaik A, Offerman MPJ. Keys to successful implementation of routine symptom monitoring in head and neck oncology with 'Healthcare Monitor' and patients' perspectives of quality of care. *HEAD AND NECK-JOURNAL FOR THE SCIENCES AND SPECIALTIES OF THE HEAD AND NECK* 2020; **42**: 3590–600.

61 Drott J, Vilhelmsson M, Kjellgren K, Bertero C. Experiences With a Self-Reported Mobile Phone-Based System Among Patients With Colorectal Cancer: A Qualitative Study. *JMIR mHealth and uHealth* 2016; **4**: 182–90.

62 Duineveld LAM, Wieldraaijer T, van Asselt KM, *et al.* Improving care after colon cancer treatment in The Netherlands, personalised care to enhance quality of life (I CARE study): study protocol for a randomised controlled trial. *Trials* 2015; **16**. DOI:10.1186/s13063-015-0798-7.

63 Duman-Lubberding S, van Uden-Kraan CF, Jansen F, *et al.* Feasibility of an eHealth application "OncoKompas" to improve personalized survivorship cancer care. *Supportive care in cancer : official journal of the Multinational Association of Supportive Care in Cancer* 2016; **24**: 2163–71.

64 Duman-Lubberding S, van Uden-Kraan CF, Jansen F, *et al.* Durable usage of patient-reported outcome measures in clinical practice to monitor health-related quality of life in head and neck cancer patients. *Supportive care in cancer : official journal of the Multinational Association of Supportive Care in Cancer* 2017; **25**: 3775–83.

65 Dunlop E, Ferguson A, Mueller T, *et al.* Involving Patients and Clinicians in the Design of Wireframes for Cancer Medicines Electronic Patient Reported Outcome Measures in Clinical Care: Mixed Methods Study. *JMIR formative research* 2023; **7**: e48296.

66 Dussel V, Orellana L, Holder R, Porth R, Avery M, Wolfe J. A multisite randomized controlled trial of an early palliative care intervention in children with advanced cancer: The PediQUEST Response Study Protocol. *PLOS ONE*; **17**. DOI:10.1371/journal.pone.0277212.

67 Efficace F, Patriarca A, Luppi M, *et al.* Physicians' Perceptions of Clinical Utility of a Digital Health Tool for Electronic Patient-Reported Outcome Monitoring in Real-Life Hematology Practice. Evidence From the GIMEMA-ALLIANCE Platform. *Frontiers in oncology* 2022; **12**: 826040.

68 Egbring M, Far E, Roos M, *et al.* A mobile app to stabilize daily functional activity of breast cancer patients in collaboration with the physician: a randomized controlled clinical trial. *Journal of medical Internet research* 2016; **18**: e238.

69 Einstein DJ, Patil D, Chipman J, *et al.* Expanded Prostate Cancer Index Composite-26 (EPIC-26) Online: Validation of an Internet-Based Instrument for Assessment of Health-Related Quality of Life After Treatment for Localized Prostate Cancer. *UROLOGY* 2019; **127**: 53–60.

70 El Shafie RA, Bougatf N, Sprave T, *et al.* Oncologic Therapy Support Via Means of a Dedicated Mobile App (OPTIMISE-1): Protocol for a Prospective Pilot Trial. *JMIR research protocols* 2018; **7**: e70.

71 Elsbernd A, Hjerming M, Visler C, *et al.* Using Cocreation in the Process of Designing a Smartphone App for Adolescents and Young Adults With Cancer: Prototype Development Study. *JMIR formative research* 2018; **2**: e23.

72 Erharter A, Giesinger J, Kemmler G, *et al.* Implementation of computer-based quality-of-life monitoring in brain tumor outpatients in routine clinical practice. *Journal of pain and symptom management* 2010; **39**: 219–29.

73 Erickson N, Schinkoethe T, Eckhardt C, *et al.* Patient-reported outcome measures obtained via E-Health tools ease the assessment burden and encourage patient participation in cancer care (PaCC Study). *Supportive care in cancer : official journal of the Multinational Association of Supportive Care in Cancer* 2021; **29**: 7715–24.

74 Fernández-Méndez R, Rastall RJ, Sage WA, *et al.* Quality improvement of neuro-oncology services: integrating the routine collection of patient-reported, health-related quality-of-life measures. *Neuro-oncology practice* 2019; **6**: 226–36.

75 Ferrè F, Rosis S, Murante AM, *et al.* Systematic and continuous collection of patient-reported outcomes and experience in women with cancer undergoing mastectomy and immediate breast reconstruction: a study protocol for the Tuscany Region (Italy). *BMJ open* 2021; **11**: e042235.

76 Ferrua M, Minvielle E, Fourcade A, *et al.* How to Design a Remote Patient Monitoring System? A French Case Study. *BMC health services research* 2020; **20**. DOI:10.1186/s12913-020-05293-4.

77 Fishbein JN, Nisotel LE, MacDonald JJ, *et al.* Mobile application to promote adherence to oral chemotherapy and symptom management: a protocol for design and development. *JMIR Research protocols* 2017; **6**: e6198.

78 Fjell M, Langius-Eklof A, Nilsson M, Wengstrom Y, Sundberg K. Reduced symptom burden with the support of an interactive app during neoadjuvant chemotherapy for breast cancer - A randomized controlled trial. *BREAST* 2020; **51**: 85–93.

79 Fortner BV, Schwartzberg LS, Stepanski EJ, Houts AC. Symptom Burden for Patients with Metastatic Colorectal Cancer Treated with First-Line FOLFOX or FOLFIRI with and Without Bevacizumab in the Community Setting. *Supportive cancer therapy* 2007; **4**: 233–40.

80 Frasquilho D, Matias R, Gracio J, *et al.* Protocol for the Implementation and Assessment of 'MoodUP': A Stepped Care Model Assisted by a Digital Platform to Accelerate Access to Mental Health Care for Cancer Patients Amid the COVID-19 Pandemic. *INTERNATIONAL JOURNAL OF ENVIRONMENTAL RESEARCH AND PUBLIC HEALTH* 2021; **18**. DOI:10.3390/ijerph18094629.

81 Frick MA, Vachani CC, Bach C, *et al.* Survivorship and the chronic cancer patient: Patterns in treatment-related effects, follow-up care, and use of survivorship care plans. *Cancer* 2017; **123**: 4268–76.

82 Frick MA, Vachani CC, Hampshire MK, *et al.* Survivorship after lower gastrointestinal cancer: Patient-reported outcomes and planning for care. *Cancer* 2017; **123**: 1860–8.

83 Frick MA, Vachani CC, Hampshire MK, *et al.* Patient-Reported Survivorship Care Practices and Late Effects After Treatment of Hodgkin and Non-Hodgkin Lymphoma. *JCO clinical cancer informatics* 2018; **2**. DOI:10.1200/CCI.18.00015.

84 Fridriksdottir N, Ingadottir B, Skuladottir K, Zoëga S, Gunnarsdottir S. Supportive Digital Health Service During Cancer Chemotherapy: Single-Arm Before-and-After Feasibility Study. *JMIR formative research* 2023; **7**: e50550.

85 Friis RB, Hjollund NH, Pappot H, Taarnhoj GA, Vestergaard JM, Skuladottir H. Patient-Reported Outcome Measures Used in Routine Care Predict for Survival at Disease Progression in Patients With Advanced Lung Cancer. *CLINICAL LUNG CANCER* 2021; **22**: E169–79.

86 Furlong E, Darley A, Fox P, *et al.* Adaptation and Implementation of a Mobile Phone-Based Remote Symptom Monitoring System for People With Cancer in Europe. *JMIR cancer* 2019; **5**: e10813.

87 Gamper EM, Nerich V, Sztankay M, *et al.* Evaluation of Noncompletion Bias and Long-Term Adherence in a 10-Year Patient-Reported Outcome Monitoring Program in Clinical Routine. *Value in health : the journal of the International Society for Pharmacoeconomics and Outcomes Research* 2017; **20**: 610–7.

88 Garcia SF, Wortman K, Cella D, *et al.* Implementing electronic health record-integrated screening of patient-reported symptoms and supportive care needs in a comprehensive cancer center. *Cancer* 2019; **125**: 4059–68.

89 Generalova O, Roy M, Hall E, *et al.* Implementation of a cloud-based electronic patient-reported outcome (ePRO) platform in patients with advanced cancer. *Journal of patient-reported outcomes* 2021; **5**: 91.

90 Gibson F, Aldiss S, Taylor RM, *et al.* Utilization of the Medical Research Council Evaluation Framework in the Development of Technology for Symptom Management The ASyMS (c)-YG Study. *Cancer nursing* 2010; **33**: 343–52.

91 Gilbert JE, Howell D, King S, *et al.* Quality Improvement in Cancer Symptom Assessment and Control: The Provincial Palliative Care Integration Project (PPCIP). *Journal of pain and symptom management* 2012; **43**: 663–78.

92 Gilljam B-M, Nygren JM, Svedberg P, Arvidsson S. Impact of an Electronic Health Service on Child Participation in Pediatric Oncology Care: Quasiexperimental Study. *Journal of medical Internet research* 2020; **22**: e17673.

93 Girgis A, Bamgboje-Ayodele A, Rincones O, *et al.* Stepping into the real world: a mixed-methods evaluation of the implementation of electronic patient reported outcomes in routine lung cancer care. *JOURNAL OF PATIENT-REPORTED OUTCOMES*; **6**. DOI:10.1186/s41687-022-00475-6.

94 Girgis A, Durcinoska I, Arnold A, *et al.* Web-Based Patient-Reported Outcome Measures for Personalized Treatment and Care (PROMPT-Care): Multicenter Pragmatic Nonrandomized Trial. *Journal of medical Internet research* 2020; **22**. DOI:10.2196/19685.

95 Girgis A, Durcinoska I, Levesque JV, *et al.* eHealth System for Collecting and Utilizing Patient Reported Outcome Measures for Personalized Treatment and Care (PROMPT-Care) Among Cancer Patients: Mixed Methods Approach to Evaluate Feasibility and Acceptability. *Journal of medical Internet research* 2017; **19**. DOI:10.2196/jmir.8360.

96 Girgis A, Delaney GP, Arnold A, *et al.* Development and Feasibility Testing of PROMPT-Care, an eHealth System for Collection and Use of Patient-Reported Outcome Measures for Personalized Treatment and Care: A Study Protocol. *JMIR research protocols* 2016; **5**: e227.

97 Girgis A, Durcinoska I, Gerges M, *et al.* Study protocol for a controlled trial of an eHealth system utilising patient reported outcome measures for personalised treatment and care: PROMPT-Care 2.0. *BMC cancer* 2018; **18**: 845.

98 Gomaa S, Posey J, Bashir B, *et al.* Feasibility of a Text Messaging-Integrated and Chatbot-Interfaced Self-Management Program for Symptom Control in Patients With Gastrointestinal Cancer Undergoing Chemotherapy: Pilot Mixed Methods Study. *JMIR formative research* 2023; **7**: e46128.

99 Graetz I, Hu X, Curry R, A. V, G. A. S. Mobile application to support oncology patients during treatment on patient outcomes: Evidence from a randomized controlled trial. *Cancer medicine* DOI:10.1002/cam4.5351.

100 Graetz I, McKillop CN, Stepanski E, Vidal GA, Anderson JN, Schwartzberg LS. Use of a web-based app to improve breast cancer symptom management and adherence for aromatase inhibitors: a randomized controlled feasibility trial. *Journal of cancer survivorship : research and practice* 2018; **12**: 431–40.

101 Graf J, Sickenberger N, Brusniak K, *et al.* Implementation of an Electronic Patient-Reported Outcome App for Health-Related Quality of Life in Breast Cancer Patients: Evaluation and Acceptability Analysis in a Two-Center Prospective Trial. *Journal of medical Internet research* 2022; **24**. DOI:10.2196/16128.

102 Grimsbo GH, Engelsrud GH, Ruland CM, Finset A. Cancer patients' experiences of using an Interactive Health Communication Application (IHCA). *INTERNATIONAL JOURNAL OF QUALITATIVE STUDIES ON HEALTH AND WELL-BEING* 2012; **7**. DOI:10.3402/qhw.v7i0.15511.

103 Gustavell T, Langius-Eklöf A, Wengström Y, Segersvärd R, Sundberg K. Development and Feasibility of an Interactive Smartphone App for Early Assessment and Management of Symptoms Following Pancreaticoduodenectomy. *Cancer nursing* 2019; **42**: E1–10.

104 Gustavell T, Sundberg KK, Langius-Eklof A. Using an Interactive App for Symptom Reporting and Management Following Pancreatic Cancer Surgery to Facilitate Person-Centered Care: Descriptive Study. *JMIR mHealth and uHealth* 2020; **8**. DOI:10.2196/17855.

105 Handa S, Okuyama H, Yamamoto H, Nakamura S, Kato Y. Effectiveness of a Smartphone Application as a Support Tool for Patients Undergoing Breast Cancer Chemotherapy: A Randomized Controlled Trial. *Clinical breast cancer* 2020; **20**: 201–8.

106 Harrison NJ, Lopez AA, Shroder MM, *et al.* Collection and Utilization of Patient-Reported Outcome Measures in a Colorectal Surgery Clinic. *The Journal of surgical research* 2022; **280**: 515–25.

107 Hassett MJ, Wong S, Osarogiagbon RU, *et al.* Implementation of patient-reported outcomes for symptom management in oncology practice through the SIMPRO research consortium: a protocol for a pragmatic type II hybrid effectiveness-implementation multi-center cluster-randomized stepped wedge trial. *Trials* 2022; **23**. DOI:10.1186/s13063-022-06435-1.

108 Hassett MJ, Cronin C, Tsou TC, *et al.* eSyM: An Electronic Health Record-Integrated Patient-Reported Outcomes-Based Cancer Symptom Management Program Used by Six Diverse Health Systems. *JCO clinical cancer informatics* 2022; **6**: e2100137.

109 Hauth F, Bizu V, App R, *et al.* Electronic Patient-Reported Outcome Measures in Radiation Oncology: Initial Experience After Workflow Implementation. *JMIR mHealth and uHealth* 2019; **7**. DOI:10.2196/12345.

110 Hea E, Wu J, Dietzen L, Harralson T, Boudreaux ED. The Polaris Oncology Survivorship Transition (POST) system: a patient-and provider-driven cancer survivorship planning program. *Journal of oncology navigation & survivorship* 2016; **7**: 11.

111 Helissey C, Parnot C, Rivière C, *et al.* Effectiveness of electronic patient reporting outcomes, by a digital telemonitoring platform, for prostate cancer care: the Protecty study. *FRONTIERS IN DIGITAL HEALTH*; **5**. DOI:10.3389/fdgth.2023.1104700.

112 Hess LM, Cui ZL, Wu Y, *et al.* Patient Experience After Receiving a Diagnosis of Gastric Cancer in the USA. *Journal of gastrointestinal cancer* 2018; **49**: 25–34.

113 Hochstenbach LMJ, Zwakhalen SMG, Courtens AM, van Kleef M, Witte LP. Feasibility of a mobile and web-based intervention to support self-management in outpatients with cancer pain. *European journal of oncology nursing : the official journal of European Oncology Nursing Society* 2016; **23**: 97–105.

114 Holch P, Warrington L, Bamforth LCA, *et al.* Development of an integrated electronic platform for patient self-report and management of adverse events during cancer treatment. *Annals of oncology : official journal of the European Society for Medical Oncology* 2017; **28**: 2305–11.

115 Holch P, Pini S, Henry AM, *et al.* eRAPID electronic patient self-Reporting of Adverse-events: Patient Information and aDvice: a pilot study protocol in pelvic radiotherapy. *Pilot and feasibility studies* 2018; **4**: 110.

116 Holzner B, Giesinger JM, Pinggera J, *et al.* The Computer-based Health Evaluation Software (CHES): a software for electronic patient-reported outcome monitoring. *BMC medical informatics and decision making* 2012; **12**: 126.

117 Hou IC, Lin HY, Shen SH, *et al.* Quality of Life of Women After a First Diagnosis of Breast Cancer Using a Self-Management Support mHealth App in Taiwan: Randomized Controlled Trial. *JMIR mHealth and uHealth* 2020; **8**. DOI:10.2196/17084.

118 Howell D, Rosberger Z, Mayer C, *et al.* Personalized symptom management: a quality improvement collaborative for implementation of patient reported outcomes (PROs) in 'real-world' oncology multisite practices. *Journal of patient-reported outcomes* 2020; **4**: 47.

119 Huberts AS, Clarijs ME, Pastoor H, van Rosmalen M, Koppert LB. Sexual well-being in patients with early-stage breast cancer at 1- and 2-year follow-up. *The journal of sexual medicine* 2023; **20**: 507–14.

120 Huelster H, Zemp L, Turner K, *et al.* Mobile Postoperative Symptom Intervention Tool and Biometric Monitoring After Radical Cystectomy: Pilot Study Evaluating Feasibility, Usability, and Potential Utility. *JOURNAL OF UROLOGY*; **209**: 410–9.

121 Iivanainen S, Baird A, Balas B, *et al.* Assessing the impact of digital patient monitoring on health outcomes and healthcare resource usage in addition to the feasibility of its combination with at-home treatment, in participants receiving systemic anticancer treatment in clinical practice: protocol for an interventional, open-label, multicountry platform study (ORIGAMA). *BMJ OPEN*; **13**. DOI:10.1136/bmjopen-2022-063242.

122 Iivanainen S, Ravichandra R, Jekunen A, *et al.* ePRO symptom follow-up of colorectal cancer patients receiving oxaliplatin-based adjuvant chemotherapy is feasible and enhances the quality of patient care: a prospective multicenter study. *JOURNAL OF CANCER RESEARCH AND CLINICAL ONCOLOGY* 2023; published online Feb 21. DOI:10.1007/s00432-023-04622-4.

123 Isakoff S, Said M, Kwak A, *et al.* Feasibility of introducing a smartphone navigation application into the care of breast cancer patients (The FIONA Study). *BREAST CANCER RESEARCH AND TREATMENT* 2023; published online April 27. DOI:10.1007/s10549-023-06918-y.

124 J Weis, LR Wolf, M Boerries, D Kassahn, M Boeker, C Dresch. Identification of the Needs and Preferences of Patients With Cancer for the Development of a Clinic App: Qualitative Study. *JMIR cancer* 2023; **9**. DOI:10.2196/40891.

125 Jones HV, Smith H, Cooksley T, *et al.* Checklists for Complications During Systemic Cancer Treatment Shared by Patients, Friends, and Health Care Professionals: Prospective Interventional Cohort Study. *JMIR mHealth and uHealth* 2020; **8**: e19225.

126 Judson TJ, Bennett AV, Rogak LJ, *et al.* Feasibility of Long-Term Patient Self-Reporting of Toxicities From Home via the Internet During Routine Chemotherapy. *Journal of clinical oncology : official journal of the American Society of Clinical Oncology* 2013; **31**: 2580-+.

127 Jung M, Lee S, Kim J, *et al.* A Mobile Technology for Collecting Patient-Reported Physical Activity and Distress Outcomes: Cross-Sectional Cohort Study. *JMIR mHealth and uHealth* 2020; **8**. DOI:10.2196/17320.

128 K Ala-Aldeen, N Stones, D Woolf, *et al.* The use of volunteers to implement electronic patient reported outcomes in lung cancer outpatient clinics. *Technical innovations & patient support in radiation oncology* 2018; **7**: 11–6.

129 Kanakubo A, Mizuno M, Asano Y, Inoue Y. Acceptability to making a self-assessment using a tablet computer and health-related quality of life in ambulatory breast cancer patients. *Asia-Pacific journal of oncology nursing* 2022; **9**: 105–12.

130 Kapoor A, Nambisan P. Usability and acceptance evaluation of ACESO: a Web-based breast cancer survivorship tool. *Journal of cancer survivorship : research and practice* 2018; **12**: 316–25.

131

Karras BT, Wolpin S, Lober WB, Bush N, Fann JR, Berry DL. Electronic Self-report Assessment--Cancer (ESRA-C): Working towards an integrated survey system. *Studies in health technology and informatics* 2006; **122**: 514–8.

132 Karsten MM, Roehle R, Albers S, *et al.* Real-world reference scores for EORTC QLQ-C30 and EORTC QLQ-BR23 in early breast cancer patients. *European journal of cancer (Oxford, England : 1990)* 2022; **163**: 128–39.

133 Karsten MM, Kühn F, Pross T, *et al.* PRO B: evaluating the effect of an alarm-based patient-reported outcome monitoring compared with usual care in metastatic breast cancer patients-study protocol for a randomised controlled trial. *Trials* 2021; **22**: 666.

134 Katzel JA, Van Den Eeden SK, Liu R, *et al.* Real-World Use of Electronic Patient-Reported Outcome (ePRO) Tools Integrated in the Electronic Medical Record During Radiation Therapy for Head and Neck Cancer: Feasibility Study. *The Permanente journal* 2023; **27**: 60–7.

135 Kearney N, Kidd L, Miller M, *et al.* Utilising handheld computers to monitor and support patients receiving chemotherapy: results of a UK-based feasibility study. *Supportive care in cancer : official journal of the Multinational Association of Supportive Care in Cancer* 2006; **14**: 742–52.

136 Kearney N, McCann L, Norrie J, *et al.* Evaluation of a mobile phone-based, advanced symptom management system (ASyMS©) in the management of chemotherapy-related toxicity. *Supportive care in cancer : official journal of the Multinational Association of Supportive Care in Cancer* 2009; **17**: 437–44.

137 Kelleher SA, Somers TJ, Locklear T, Crosswell AD, Abernethy AP. Using Patient Reported Outcomes in Oncology Clinical Practice. *Scandinavian journal of pain* 2016; **13**: 6–11.

138 Kim J, Lim S, Min YH, *et al.* Depression screening using daily mental-health ratings from a smartphone application for breast cancer patients. *Journal of medical Internet research* 2016; **18**: e5598.

139 Klasnja P, Hartzler A, Powell C, Phan G, Pratt W. Health weaver mobile: designing a mobile tool for managing personal health information during cancer care. In: AMIA Annual Symposium Proceedings. American Medical Informatics Association: 392.

140 Knoerl R, Dudley WN, Smith G, Bridges C, Kanzawa-Lee G, Smith EML. Pilot Testing a Web-Based System for the Assessment and Management of Chemotherapy-Induced Peripheral Neuropathy. *CIN-COMPUTERS INFORMATICS NURSING* 2017; **35**: 201–11.

141 Knoerl R, Gray E, Stricker C, *et al.* Electronic versus paper-pencil methods for assessing chemotherapy-induced peripheral neuropathy. *Supportive care in cancer : official journal of the Multinational Association of Supportive Care in Cancer* 2017; **25**: 3437–46.

142 Krogstad H, Brunelli C, Sand K, *et al.* Development of EirV3: A Computer-Based Tool for Patient-Reported Outcome Measures in Cancer. *JCO clinical cancer informatics* 2017; **1**: 1–14.

143 Krogstad H, Sundt-Hansen SM, Hjermstad MJ, *et al.* Usability testing of EirV3-a computer-based tool for patient-reported outcome measures in cancer. *Supportive care in cancer : official journal of the Multinational Association of Supportive Care in Cancer* 2019; **27**: 1835–44.

144 Kudel I, Perry T. Communicating Treatment-Related Symptoms Using Passively Collected Data and Satisfaction/Loyalty Ratings: Exploratory Study. *JMIR CANCER*; **8**. DOI:10.2196/29292.

145 Kuo JC, Graham DM, Salvarrey A, *et al.* A randomized trial of the electronic Lung Cancer Symptom Scale for quality-of-life assessment in patients with advanced non-small-cell lung cancer. *Current oncology (Toronto, Ont)* 2020; **27**: e156–62.

146 Langius-Eklöf A, Christiansen M, Lindström V, *et al.* Adherence to Report and Patient Perception of an Interactive App for Managing Symptoms During Radiotherapy for Prostate Cancer: Descriptive Study of Logged and Interview Data. *JMIR cancer* 2017; **3**. DOI:10.2196/cancer.7599.

147 Langius-Eklof A, Crafoord MT, Christiansen M, Fjell M, Sundberg K. Effects of an interactive mHealth innovation for early detection of patient-reported symptom distress with focus on participatory care: protocol for a study based on prospective, randomised, controlled trials in patients with prostate and breast cancer. *BMC cancer* 2017; **17**. DOI:10.1186/s12885-017-3450-y.

148 Lapen K, Sabol C, Tin AL, *et al.* Development and Pilot Implementation of a Remote Monitoring System for Acute Toxicity Using Electronic Patient-Reported Outcomes for Patients Undergoing Radiation Therapy for Breast Cancer. *International journal of radiation oncology, biology, physics* 2021; **111**: 979–91.

149 Leahy A, La Schwartz L, Y. M. R, B. B. B, J. E. A, R. B. Electronic symptom monitoring in pediatric patients hospitalized for chemotherapy. *Cancer* 2021; **127**: 2980–9.

150 Lee JH, Jeong JH, Ji W, *et al.* Comparative effectiveness of smartphone healthcare applications for improving quality of life in lung cancer patients: study protocol. *BMC PULMONARY MEDICINE* 2022; **22**. DOI:10.1186/s12890-022-01970-8.

151 Lee M, Kang D, Kang E, *et al.* Efficacy of the PRO-CTCAE mobile application for improving patient participation in symptom management during cancer treatment: a randomized controlled trial. *Supportive care in cancer : official journal of the Multinational Association of Supportive Care in Cancer* 2023; **31**: 321.

152 Lehmann J, Wintner LM, Sztankay M, *et al.* Patient-reported outcomes and psycho-oncological screening in hematology: a practical example of routine electronic monitoring. *MEMO-MAGAZINE OF EUROPEAN MEDICAL ONCOLOGY* 2020; **13**: 285–93.

153 Lehmann J, Ligt KM, Tipelius S, *et al.* Adherence to Patient-Reported Symptom Monitoring and Subsequent Clinical Interventions for Patients With Multiple Myeloma in Outpatient Care: Longitudinal Observational Study. *Journal of medical Internet research* 2023; **25**: e46017.

154 Leung YW, Li M, Devins G, *et al.* Routine screening for suicidal intention in patients with cancer. *Psycho-oncology* 2013; **22**: 2537–45.

155 Licht T, Nickels A, Rumpold G, Holzner B, Riedl D. Evaluation by electronic patient-reported outcomes of cancer survivors' needs and the efficacy of inpatient cancer rehabilitation in different tumor entities. *Supportive care in cancer : official journal of the Multinational Association of Supportive Care in Cancer* 2021; **29**: 5853–64.

156 Lim S, Ke Y, Mok N, *et al.* Factors associated with distress and the impact of distress on acute health-care service utilization among patients diagnosed with breast and gynecological cancers. *PALLIATIVE & SUPPORTIVE CARE* 2023; published online Jan 9. DOI:10.1017/S1478951522001444.

157 Lin HC, Wu HC, Chang CH, Li TC, Liang WM, Wang JY. A real time online assessment system with modelized architecture on clinical infometrics for patient reported outcomes of prostate cancer. *COMPUTER METHODS AND PROGRAMS IN BIOMEDICINE* 2012; **106**: 249–59.

158 Lin TH, Wang YM, Huang CY. Effects of a mobile oral care app on oral mucositis, pain, nutritional status, and quality of life in patients with head and neck cancer: A quasi-experimental study. *INTERNATIONAL JOURNAL OF NURSING PRACTICE* 2022; **28**. DOI:10.1111/ijn.13042.

159 Lobach DF, Johns EB, Halpenny B, *et al.* Increasing Complexity in Rule-Based Clinical Decision Support: The Symptom Assessment and Management Intervention. *JMIR medical informatics* 2016; **4**: 62–75.

160 Lombi L, Alfieri S, Brunelli C. ?Why should I fill out this questionnaire?? A qualitative study of cancer patients? perspectives on the integration of e-PROMs in routine clinical care. *EUROPEAN JOURNAL OF ONCOLOGY NURSING*; **63**. DOI:10.1016/j.ejon.2023.102283.

161 Lopes A, Colomer-Lahiguera S, Darnac C, *et al.* Development of an eHealth-enhanced model of care for the monitoring and management of immune-related adverse events in patients treated with immune checkpoint inhibitors. *SUPPORTIVE CARE IN CANCER*; **31**. DOI:10.1007/s00520-023-07934-w.

162 Lucas AR, Bass MB, Rothrock NE, *et al.* Development of an eHealth System to Capture and Analyze Patient Sensor and Self-Report Data: Mixed-Methods Assessment of Potential Applications to Improve Cancer Care Delivery. *JMIR medical informatics* 2018; **6**: 138–50.

163 Maas A, Maurice-Stam H, van den Heuvel M, *et al.* Monitoring health related quality of life in survivorship care of young adult survivors of childhood cancer using web-based patient-reported outcome measures: survivors' and health care practitioners' perspectives on the KLIK method. *QUALITY OF LIFE RESEARCH* 2023; published online Aug 24. DOI:10.1007/s11136-023-03504-z.

164 Macpherson CF, Stegenga K, Erickson JM, *et al.* Adolescents and Young Adults with Cancer Using a Symptom Heuristics App: Provider Perceptions and Actions. *JOURNAL OF ADOLESCENT AND YOUNG ADULT ONCOLOGY* 2020; **9**: 579–85.

165 Maguire R, Ream E, Richardson A, *et al.* Development of a Novel Remote Patient Monitoring System The Advanced Symptom Management System for Radiotherapy to Improve the Symptom Experience of Patients With Lung Cancer Receiving Radiotherapy. *Cancer nursing* 2015; **38**: E37–47.

166 Maguire R, Fox PA, McCann L, *et al.* The eSMART study protocol: a randomised controlled trial to evaluate electronic symptom management using the advanced symptom management system (ASyMS) remote technology for patients with cancer. *BMJ open* 2017; **7**: e015016.

167 Majumdar JR, Fromkin JB, Yermal SJ, Fatata-Haim AM, Barton-Burke M, Jairath NN. Research Electronic Data Capture (REDCap) in an outpatient oncology surgery setting to securely email, collect, and manage survey data. *Journal of advanced nursing* 2023. DOI:10.1111/jan.15983.

168 Mamguem Kamga A, Di Martino C, Anota A, *et al.* Impact of routine assessment of health-related quality of life coupled with therapeutic information on compliance with endocrine therapy in patients with non-metastatic breast cancer: protocol for a randomized controlled trial. *Trials* 2020; **21**: 527.

169 Mark TL, Johnson G, Fortner B, Ryan K. The benefits and challenges of using computer-assisted symptom assessments in oncology clinics: Results of a qualitative assessment. *Technology in cancer research & treatment* 2008; **7**: 401–5.

170 Mary NN, Hoyer S, Friis S, *et al.* The Danish Prostate Cancer Database. *CLINICAL EPIDEMIOLOGY* 2016; **8**: 649–53.

171 Masiero M, Filipponi C, Pizzoli S, *et al.* Usability Testing of a New Digital Integrated Health Ecosystem (PainRELife) for the Clinical Management of Chronic Pain in Patients With Early Breast Cancer: Protocol for a Pilot Study. *JMIR RESEARCH PROTOCOLS* 2023; **12**. DOI:10.2196/41216.

172 Matthijs de Wit L, van Uden-Kraan CF, Lissenberg-Witte BI, *et al.* Adoption and implementation of a web-based self-management application 'Oncokompas' in routine cancer care: a national pilot study. *Supportive care in cancer : official journal of the Multinational Association of Supportive Care in Cancer* 2019; **27**: 2911–20.

173 McGee MR, Gray P. A handheld chemotherapy symptom management system: results from a preliminary outpatient field trial. *Health Informatics Journal* 2005; **11**: 243–58.

174 McNeill NA, Kors WA, Bosscha MI, *et al.* Feasibility of RetinoQuest: e-health application to facilitate and improve additional care for retinoblastoma survivors. *Journal of cancer survivorship : research and practice* 2017; **11**: 683–90.

175 Medina JC, Flix-Valle A, Rodríguez-Ortega A, Hernández-Ribas R, Frutos ML, Ochoa-Arnedo C. ICOnnecta't: Development and Initial Results of a Stepped Psychosocial eHealth Ecosystem to Facilitate Risk Assessment and Prevention of Early Emotional Distress in Breast Cancer Survivors' Journey. *Cancers* 2022; **14**. DOI:10.3390/cancers14040974.

176 Mehdizadeh H, Asadi F, Emami H, Mehrvar A, Nazemi E. An mHealth Self-management System for Support Children With Acute Lymphocytic Leukemia and Their Caregivers: Qualitative Co-design Study. *JMIR FORMATIVE RESEARCH*; **6**. DOI:10.2196/36721.

177 Mehdizadeh H, Asadi F, Nazemi E, Mehrvar A, Yazdanian A, Emami H. A Mobile Self-Management App (CanSelfMan) for Children With Cancer and Their Caregivers: Usability and Compatibility Study. *JMIR PEDIATRICS AND PARENTING* 2023; **6**. DOI:10.2196/43867.

178 Meryk A, Kropshofer G, Hetzer B, *et al.* Implementation of daily patient-reported outcome measurements to support children with cancer. *Pediatric blood & cancer* 2021; **68**. DOI:10.1002/pbc.29279.

179 Meryk A, Kropshofer G, Hetzer B, *et al.* Bridging the gap in outpatient care: Can a daily patient-reported outcome measure help? *Cancer reports (Hoboken, NJ)* 2022; **5**: e1421.

180 Merz A, Mohamed A, Corbett C, *et al.* A single-site pilot feasibility randomized trial of a supportive care mobile application intervention for patients with advanced cancer and caregivers. *Supportive care in cancer : official journal of the Multinational Association of Supportive Care in Cancer* 2022; **30**: 7853–61.

181 Middeke M, Bauhofer A, Kopp I, Koller M. Computerized visualization of quality of life data of individual cancer patients--the QoL-Profiler. *Inflammation research : official journal of the European Histamine Research Society . [et al]* 2004; **53 Suppl 2**: S175-8.

182 Miller M, Roxburgh CS, McCann L, *et al.* Development of a Remote Monitoring Application to Improve Care and Support Patients in the First 30 Days Following Colorectal Cancer Surgery. *Seminars in oncology nursing* 2020; **36**. DOI:10.1016/j.soncn.2020.151086.

183 Min YH, Lee JW, Shin YW, *et al.* Daily Collection of Self-Reporting Sleep Disturbance Data via a Smartphone App in Breast Cancer Patients Receiving Chemotherapy: A Feasibility Study. *Journal of medical Internet research* 2014; **16**: 87–100.

184 Mir O, Ferrua M, Fourcade A, *et al.* Digital remote monitoring plus usual care versus usual care in patients treated with oral anticancer agents: the randomized phase 3 CAPRI trial. *Nature medicine* 2022; **28**: 1224-+.

185 Mirkovic J, Kaufman R. Supporting Cancer Patients in Illness Management: Usability Evaluation of a Mobile App. *JMIR mHealth and uHealth* 2014; **2**. DOI:10.2196/mhealth.3359.

186 Misplon S, Marneffe W, Himpe U, Hellings J, Demedts I. Evaluation of the implementation of Value-Based Healthcare with a weekly digital follow-up of lung cancer patients in clinical practice. *European journal of cancer care* 2022; **31**: e13653.

187 Mlakar I, Safran V, Hari D, *et al.* Multilingual Conversational Systems to Drive the Collection of Patient-Reported Outcomes and Integration into Clinical Workflows. *SYMMETRY-BASEL* 2021; **13**. DOI:10.3390/sym13071187.

188 Mohseni M, Ayatollahi H, Arefpour A. Electronic patient-reported outcome (ePRO) application for patients with prostate cancer. *PLOS ONE*; **18**. DOI:10.1371/journal.pone.0289974.

189 Mooney K, Whisenant MS, Beck SL. Symptom Care at Home A Comprehensive and Pragmatic PRO System Approach to Improve Cancer Symptom Care. *Medical care* 2019; **57**: S66–72.

190 Mouillet G, Falcoz A, Thiery-Vuillemin A, *et al.* Feasibility of health-related quality of life (HRQoL) assessment for cancer patients using electronic patient-reported outcome (ePRO) in daily clinical practice: a French experience. *QUALITY OF LIFE RESEARCH* 2019; **28**: S120–S120.

191 Nahum JL, Fu MR, Scagliola J, *et al.* Real-time electronic patient evaluation of lymphedema symptoms, referral, and satisfaction: a cross-sectional study. *mHealth* 2021; **7**: 20.

192 Naughton MJ, Salani R, Peng J, *et al.* Feasibility of implementing a text-based symptom-monitoring program of endometrial, ovarian, and breast cancer patients during treatment. *Quality of life research : an international journal of quality of life aspects of treatment, care and rehabilitation* 2021; **30**: 3241–54.

193 Nipp RD, El-Jawahri A, Ruddy M, *et al.* Pilot randomized trial of an electronic symptom monitoring intervention for hospitalized patients with cancer. *Annals of oncology : official journal of the European Society for Medical Oncology* 2019; **30**: 274–80.

194 Nipp RD, Horick NK, Deal R, *et al.* Differential effects of an electronic symptom monitoring intervention based on the age of patients with advanced cancer. *Annals of oncology : official journal of the European Society for Medical Oncology* 2020; **31**: 123–30.

195 Nipp RD, Horick NK, Qian CL, *et al.* Effect of a Symptom Monitoring Intervention for Patients Hospitalized With Advanced Cancer A Randomized Clinical Trial. *JAMA ONCOLOGY* 2022; **8**: 571–8.

196 Nordhausen T, Lampe K, Vordermark D, *et al.* An implementation study of electronic assessment of patient-reported outcomes in inpatient radiation oncology. *JOURNAL OF PATIENT-REPORTED OUTCOMES*; **6**. DOI:10.1186/s41687-022-00478-3.

197 Nyman MH, Frank C, Langius-Eklof A, Blomberg K, Sundberg K, Wengstrom Y. Patients' Perspective on Participation in Care With or Without the Support of a Smartphone App During Radiotherapy for Prostate Cancer: Qualitative Study. *JMIR mHealth and uHealth* 2017; **5**. DOI:10.2196/mhealth.6829.

198 Oakley-Girvan I, Yunis R, Fonda S, *et al.* Usability evaluation of mobile phone technologies for capturing cancer patient-reported outcomes and physical functions. *DIGITAL HEALTH*; **9**. DOI:10.1177/20552076231186515.

199 Oerlemans S, Arts LPJ, Kieffer JM, *et al.* Web-Based Return of Individual Patient-Reported Outcome Results Among Patients With Lymphoma: Randomized Controlled Trial. *Journal of medical Internet research* 2021; **23**. DOI:10.2196/27886.

200 Olson RA, Howard F, Lapointe V, *et al.* Provincial development of a patient-reported outcome initiative to guide patient care, quality improvement, and research. *Healthcare management forum* 2018; **31**: 13–7.

201 Osawa T, Fujii Y, Kimura G, *et al.* Electronic patient-reported outcome (e-PRO) monitoring for adverse event management during cabozantinib treatment in patients with advanced renal cell carcinoma: protocol for a three-arm, randomised, multicentre phase II trial (e-PRO vs paper-PRO or usual care). *BMJ open* 2023; **13**: e070275.

202 Ossowski S, Kammerer A, Stram D, Piazza-DeLap L, Basch E, Katzel JA. Patient-Reported Outcomes Integrated Within an Electronic Medical Record in Patients With Head and Neck Cancer. *JCO clinical cancer informatics* 2021; **5**: 842–8.

203 Paladino AJ, Anderson JN, Krukowski RA, *et al.* THRIVE study protocol: a randomized controlled trial evaluating a web-based app and tailored messages to improve adherence to adjuvant endocrine therapy among women with breast cancer. *BMC health services research* 2019; **19**. DOI:10.1186/s12913-019-4588-x.

204 Palos GR, Suarez-Almazor ME. Launching an Electronic Patient-Reported Outcomes Initiative in Real-Time Clinical Practice. *Journal of the National Cancer Institute Monographs* 2021; **2021**: 23–30.

205 Pantiora E, Hedman L-C, Aristokleous I, Sjökvist O, Karakatsanis A, Schiza A. Effect of mode of delivery of patient reported outcomes in patients with breast disease: a randomised controlled trial. *International journal of surgery (London, England)* 2023; **110**: 176–82.

206 Pappot H, Baeksted CW, Nissen A, *et al.* Clinical effects of assessing electronic patient-reported outcomes monitoring symptomatic toxicities during breast cancer therapy: a nationwide and population-based study. *BREAST CANCER* 2021; **28**: 1096–9.

207 Parker B, Rajapakshe R, Moldovan A, Araujo C, Crook J. An Internet-Based Means of Monitoring Quality of Life in Post-Prostate Radiation Treatment: A Prospective Cohort Study. *JMIR research protocols* 2015; **4**: e115.

208 Passardi A, Rizzo M, Maines F, *et al.* Optimisation and validation of a remote monitoring system (Onco-TreC) for home-based management of oral anticancer therapies: an Italian multicentre feasibility study. *BMJ open* 2017; **7**: e014617.

209 Peltola MK, Poikonen-Saksela P, Mattson J, Parkkari T. A Novel Digital Patient-Reported Outcome Platform (Noona) for Clinical Use in Patients With Cancer: Pilot Study Assessing Suitability. *JMIR formative research* 2021; **5**: e16156.

210 Petersen MA, Aaronson NK, Arraras JI, *et al.* The EORTC CAT Core-The computer adaptive version of the EORTC QLQ-C30 questionnaire. *European journal of cancer (Oxford, England : 1990)* 2018; **100**: 8–16.

211 Pompili C, Boele F, Absolom K, *et al.* Patients' views of routine quality of life assessment following a diagnosis of early-stage non-small cell lung cancer. *Interactive cardiovascular and thoracic surgery* 2020; **31**: 324–30.

212 Pradhan A, Dwivedi P, Pareek P, *et al.* Electronic-PRO Measures for adverse events Of Treatment In ONcology (E-PROMOTION). *International journal of medical informatics* 2024; **182**: 105305.

213 Prasongsook N, Seetalarom K, Saichaemchan S, Udomdamrongkul K. A Pilot Study of Using Smartphone Application vs. Routine Follow-Up for Patient Care in Advanced Non-Small Cell Lung Cancer During the COVID-19 Pandemic Era. *FRONTIERS IN MEDICAL TECHNOLOGY*; **4**. DOI:10.3389/fmedt.2022.900172.

214 Prince RM, Yee AS, Parente L, *et al.* User-Centered Design of a Web-Based Tool to Support Management of Chemotherapy-Related Toxicities in Cancer Patients. *Journal of medical Internet research* 2019; **21**. DOI:10.2196/jmir.9958.

215 Rasschaert M, Helsen S, Rolfo C, van Brussel I, Ravelingien J, Peeters M. Feasibility of an interactive electronic self-report tool for oral cancer therapy in an outpatient setting. *Supportive care in cancer : official journal of the Multinational Association of Supportive Care in Cancer* 2016; **24**: 3567–71.

216 Rasschaert M, Vulsteke C, Keersmaeker S, *et al.* AMTRA: a multicentered experience of a web-based monitoring and tailored toxicity management system for cancer patients. *Supportive care in cancer : official journal of the Multinational Association of Supportive Care in Cancer* 2021; **29**: 859–67.

217 Reis J, Travado L, Scherrer A, *et al.* Digital Guardian Angel Supported by an Artificial Intelligence System to Improve Quality of Life, Well-being, and Health Outcomes of Patients With Cancer (ONCORELIEF):

Protocol for a Single Arm Prospective Multicenter Pilot Study. *JMIR RESEARCH PROTOCOLS*; **12**. DOI:10.2196/45475.

218 Requena ML, Avery M, Feraco AM, Uzal LG, Wolfe J, Dussel V. Normalization of Symptoms in Advanced Child Cancer: The PediQUEST-Response Case Study. *Journal of pain and symptom management* 2022; **63**: 548–62.

219 Rocque GB, Dionne-Odom JN, Stover D, *et al.* Evaluating the implementation and impact of navigator-supported remote symptom monitoring and management: a protocol for a hybrid type 2 clinical trial. *BMC health services research* 2022; **22**. DOI:10.1186/s12913-022-07914-6.

220 Rocque GB, Dent DN, Ingram SA, *et al.* Adaptation of Remote Symptom Monitoring Using Electronic Patient-Reported Outcomes for Implementation in Real-World Settings. *JCO oncology practice* 2022; **18**: e1943–52.

221 Rollison D, Gonzalez B, Turner K, *et al.* Examining disparities in large-scale patient-reported data capture using digital tools among cancer patients at clinical intake. *CANCER MEDICINE* 2023; published online Aug 18. DOI:10.1002/cam4.6459.

222 Rose P, Quail H, McPhelim J, Simpson M. Experiences and outcomes of lung cancer patients using electronic assessments. *Cancer Nurs Pract* 2017; **16**: 26–30.

223 Rosett HA, Locke SC, Wolf SP, *et al.* An analysis of missing items in real-world electronic patient reported outcomes data: implications for clinical care. *Supportive care in cancer : official journal of the Multinational Association of Supportive Care in Cancer* 2020; **28**: 5099–107.

224 Rotenstein LS, Agarwal A, O'Neil K, *et al.* Implementing patient-reported outcome surveys as part of routine care: lessons from an academic radiation oncology department. *Journal of the American Medical Informatics Association : JAMIA* 2017; **24**: 964–8.

225 Ruland CM, Holte HH, Roislien J, *et al.* Effects of a computer-supported interactive tailored patient assessment tool on patient care, symptom distress, and patients' need for symptom management support: a randomized clinical trial. *Journal of the American Medical Informatics Association : JAMIA* 2010; **17**: 403–10.

226 S Moradian, MK Krzyzanowska, R Maguire, *et al.* Usability Evaluation of a Mobile Phone-Based System for Remote Monitoring and Management of Chemotherapy-Related Side Effects in Cancer Patients: Mixed-Methods Study. *JMIR cancer* 2018; **4**. DOI:10.2196/10932.

227 Salimian N, Ehteshami A, Ashouri-Talouki M. Developing Ghasedak: a Mobile Application to Improve the Quality of Cancer Palliative Care. *Acta informatica medica : AIM : journal of the Society for Medical Informatics of Bosnia & Herzegovina : casopis Drustva za medicinsku informatiku BiH* 2019; **27**: 19–22.

228 Salz T, McCabe MS, Oeffinger KC, *et al.* A head and neck cancer intervention for use in survivorship clinics: a protocol for a feasibility study. *Pilot and feasibility studies* 2016; **2**: 23.

229 Salz T, Schnall RB, McCabe MS, *et al.* Incorporating multiple perspectives into the development of an electronic survivorship platform for head and neck cancer. *JCO clinical cancer informatics* 2018; **2**: 1–15.

230 Samsa GP, Wolf S, LeBlanc TW, Abernethy AP. An Exploratory Factor Analysis of the Scale Structure of the Patient Care Monitor Version 2.0. *Journal of pain and symptom management* 2016; **51**: 776-U331.

231 Schepers SA, Nicolaas SMS, Haverman L, *et al.* Real-world implementation of electronic patient-reported outcomes in outpatient pediatric cancer care. *Psycho-oncology* 2017; **26**: 951–9.

232 Schmalz O, Jacob C, Ammann J, *et al.* Digital Monitoring and Management of Patients With Advanced or Metastatic Non-Small Cell Lung Cancer Treated With Cancer Immunotherapy and Its Impact on Quality of Clinical Care: Interview and Survey Study Among Health Care Professionals and Patients. *Journal of medical Internet research* 2020; **22**. DOI:10.2196/18655.

233 Schuler MK, Trautmann F, Radloff M, *et al.* Implementation of a mobile inpatient quality of life (QoL) assessment for oncology nursing. *Supportive care in cancer : official journal of the Multinational Association of Supportive Care in Cancer* 2016; **24**: 3391–9.

234 Schuler M, Schildmann J, Trautmann F, *et al.* Cancer patients' control preferences in decision making and associations with patient-reported outcomes: a prospective study in an outpatient cancer center. *Supportive care in cancer : official journal of the Multinational Association of Supportive Care in Cancer* 2017; **25**: 2753–60.

235 Schuler M, Trautmann F, Radloff M, *et al.* Implementation and first results of a tablet-based assessment referring to patient-reported outcomes in an inpatient cancer care unit. *Zeitschrift fur Evidenz, Fortbildung und Qualitat im Gesundheitswesen* 2017; **121**: 64–72.

236 Schuler T, Back M, Hruby G, *et al.* Introducing Computed Tomography Simulation-Free and Electronic Patient-Reported Outcomes-Monitored Palliative Radiation Therapy into Routine Care: Clinical Outcomes and Implementation Experience. *Advances in radiation oncology* 2021; **6**. DOI:10.1016/j.adro.2020.100632.

237 Schunn FA, El Shafie RA, Kronsteiner D, *et al.* Oncologic treatment support via a dedicated mobile app: a prospective feasibility evaluation (OPTIMISE-1). *Strahlentherapie und Onkologie : Organ der Deutschen Rontgengesellschaft . [et al]* 2023. DOI:10.1007/s00066-023-02166-7.

238 Semple CJ, Lannon D, Qudairat E, McCaughan E, McCormac R. Development and evaluation of a holistic surgical head and neck cancer post-treatment follow-up clinic using touchscreen technology—feasibility study. *European journal of cancer care* 2018; **27**: e12809.

239 Seneviratne MG, Bozkurt S, Patel MI, *et al.* Distribution of Global Health Measures From Routinely Collected PROMIS Surveys in Patients With Breast Cancer or Prostate Cancer. *Cancer* 2019; **125**: 943–51.

240 Silva Lopes AM, Colomer-Lahiguera S, Darnac C, *et al.* Testing a Model of Care for Patients on Immune Checkpoint Inhibitors Based on Electronic Patient-Reported Outcomes: Protocol for a Randomized Phase II Controlled Trial. *JMIR research protocols* 2023; **12**: e48386.

241 Smith A, Samuel CA, McCabe SD, *et al.* Feasibility and delivery of patient-reported outcomes in clinical practice among racially diverse bladder and prostate cancer patients. *Urologic oncology* 2021; **39**. DOI:10.1016/j.urolonc.2020.06.030.

242 Snyder CF, Blackford AL, Wolff AC, Carducci MA, Herman JM, Wu AW. Feasibility and value of PatientViewpoint: a web system for patient-reported outcomes assessment in clinical practice. *Psycho-oncology* 2013; **22**: 895–901.

243 Snyder CF, Jensen R, Courtin SO, Wu AW. PatientViewpoint: a website for patient-reported outcomes assessment. *Quality of life research : an international journal of quality of life aspects of treatment, care and rehabilitation* 2009; **18**: 793–800.

244 Snyder C, Hannum SM, White S, *et al.* A PRO-cision medicine intervention to personalize cancer care using patient-reported outcomes: intervention development and feasibility-testing. *Quality of life research : an international journal of quality of life aspects of treatment, care and rehabilitation* 2022; **31**: 2341–55.

245 Sohl S, Duncan P, Thakur E, *et al.* Adaptation of a Personalized Electronic Care Planning Tool for Cancer Follow-up Care: Formative Study. *JMIR FORMATIVE RESEARCH* 2023; **7**. DOI:10.2196/41354.

246 Sprave T, Zöller D, Stoian R, *et al.* App-Controlled Treatment Monitoring and Support for Head and Neck Cancer Patients (APCOT): Protocol for a Prospective Randomized Controlled Trial. *JMIR research protocols* 2020; **9**: e21693.

247 Stabile C, Temple LK, Ancker JS, *et al.* Ambulatory cancer care electronic symptom self-reporting (ACCESS) for surgical patients: a randomised controlled trial protocol. *BMJ open* 2019; **9**: e030863.

248 Stamp E, Clarke G, Wright P, *et al.* Collection of cancer Patient Reported Outcome Measures (PROMS) to link with primary and secondary electronic care records to understand and improve long term cancer outcomes: A protocol paper. *PloS one* 2022; **17**: e0266804.

249 Stangl S, Haas K, Eichner FA, *et al.* Development and proof-of-concept of a multicenter, patient-centered cancer registry for breast cancer patients with metastatic disease-the 'Breast cancer care for patients with metastatic disease' (BRE-4-MED) registry. *Pilot and feasibility studies* 2020; **6**: 11.

250 Strachna O, Cohen MA, Allison MM, *et al.* Case study of the integration of electronic patient-reported outcomes as standard of care in a head and neck oncology practice: Obstacles and opportunities. *Cancer* 2021; **127**: 359–71.

251 Strachna O, Asan O, Stetson PD. Managing Critical Patient-Reported Outcome Measures in Oncology Settings: System Development and Retrospective Study. *JMIR medical informatics* 2022; **10**: e38483.

252 Suh S-Y, LeBlanc TW, Shelby RA, Samsa GP, Abernethy AP. Longitudinal patient-reported performance status assessment in the cancer clinic is feasible and prognostic. *Journal of oncology practice* 2011; **7**: 374–81.

253 Sundberg K, Eklof AL, Blomberg K, Isaksson AK, Wengstrom Y. Feasibility of an interactive ICT-platform for early assessment and management of patient-reported symptoms during radiotherapy for prostate cancer. *European journal of oncology nursing : the official journal of European Oncology Nursing Society* 2015; **19**: 523–8.

254 Sundberg K, Lindstrom V, Petersson LM, Langius-Eklof A. Supporting health literacy using an interactive app for symptom management during radiotherapy for prostate cancer. *Patient education and counseling* 2021; **104**: 381–6.

255 Sundberg K, Wengström Y, Blomberg K, Hälleberg-Nyman M, Frank C, Langius-Eklöf A. Early detection and management of symptoms using an interactive smartphone application (Interaktor) during radiotherapy for prostate cancer. *Supportive care in cancer : official journal of the Multinational Association of Supportive Care in Cancer* 2017; **25**: 2195–204.

256 Syrjala KL, Crouch ML, Leisenring WM, *et al.* Engagement with INSPIRE, an Online Program for Hematopoietic Cell Transplantation Survivors. *BIOLOGY OF BLOOD AND MARROW TRANSPLANTATION* 2018; **24**: 1692–8.

257 Sztankay M, Neppl L, Wintner LM, *et al.* Complementing clinical cancer registry data with patient reported outcomes: A feasibility study on routine electronic patient-reported outcome assessment for the Austrian Myelome Registry. *European journal of cancer care* 2019; **28**. DOI:10.1111/ecc.13154.

258 Taarnhoj GA, Lindberg H, Dohn LH, *et al.* Electronic reporting of patient-reported outcomes in a fragile and comorbid population during cancer therapy - a feasibility study. *Health and quality of life outcomes* 2020; **18**. DOI:10.1186/s12955-020-01480-3.

259 Takala L, Kuusinen TE, Skytta T, Kellokumpu-Lehtinen PL, Barlund M. Electronic Patient-reported Outcomes During Breast Cancer Adjuvant Radiotherapy. *Clinical breast cancer* 2021; **21**: E252–70.

260 Tang L, Pang Y, He Y, *et al.* Longitudinal study of symptom burden in outpatients with advanced cancers based on electronic Patient-Reported Outcome (ePRO) platform: a single institution, prospective study protocol. *BMJ open* 2020; **10**: e038223.

261 Taramasco C, Rimassa C, Noël R, Bravo Storm ML, Sánchez C. Co-design of a Mobile App for Engaging Breast Cancer Patients in Reporting Health Experiences: Qualitative Case Study. *Journal of medical Internet research* 2023; **25**: e45968.

262 Thestrup Hansen S, Piil K, Bak Hansen L, Ledertoug KM, Hølge-Hazelton B, Schmidt VJ. Electronic patient-reported outcome measures to enable systematic follow-up in treatment and care of women diagnosed with breast cancer: a feasibility study protocol. *BMJ open* 2022; **12**: e065110.

263 Tran C, Dicker A, Leiby B, Gressen E, Williams N, Jim H. Utilizing Digital Health to Collect Electronic Patient-Reported Outcomes in Prostate Cancer: Single-Arm Pilot Trial. *Journal of medical Internet research* 2020; **22**: e12689.

264 Trautmann F, Hentschel L, Hornemann B, *et al.* Electronic real-time assessment of patient-reported outcomes in routine care first findings and experiences from the implementation in a comprehensive cancer center. *Supportive care in cancer : official journal of the Multinational Association of Supportive Care in Cancer* 2016; **24**: 3047–56.

265 Trojan A, Leuthold N, Thomssen C, *et al.* The Effect of Collaborative Reviews of Electronic Patient-Reported Outcomes on the Congruence of Patient- and Clinician-Reported Toxicity in Cancer Patients Receiving Systemic Therapy: Prospective, Multicenter, Observational Clinical Trial. *Journal of medical Internet research* 2021; **23**: e29271.

266 Tsangaris E, Edelen M, Means J, *et al.* User-centered design and agile development of a novel mobile health application and clinician dashboard to support the collection and reporting of patient-reported outcomes for breast cancer care. *BMJ surgery, interventions, & health technologies* 2022; **4**: e000119.

267 Tsangaris E, Hyland C, Liang G, *et al.* Feasibility of implementing patient-reported outcome measures into routine breast cancer care delivery using a novel collection and reporting platform. *JAMIA open* 2023; **6**: ooad108.

268 Tsimicalis A, Le May S, Stinson J, *et al.* Linguistic Validation of an Interactive Communication Tool to Help French-Speaking Children Express Their Cancer Symptoms. *JOURNAL OF PEDIATRIC ONCOLOGY NURSING* 2017; **34**: 98–105.

269 Tumeh I, Bergerot CD, Lee D, Philip EJ, Freitas R. mHealth program for patients with advanced cancer receiving treatment in a public health hospital in Brazil. *Psycho-oncology* DOI:10.1002/pon.6059.

270 Turner K, Stover A, Tometich D, *et al.* Oncology Providers' and Professionals' Experiences With Suicide Risk Screening Among Patients With Head and Neck Cancer: A Qualitative Study. *JCO ONCOLOGY PRACTICE*; **19**: 370-+.

271 Underwood J, McCloskey S, Raldow A, *et al.* Developing a Mobile Patient-Reported Outcomes Version of the Common Terminology Criteria for Adverse Events Administration System to Capture Postradiation Toxicity in Oncology: Usability and Feasibility Study. *JMIR FORMATIVE RESEARCH*; **6**. DOI:10.2196/27775.

272 van den Brink JL, Moorman PW, Boer MF, van Bemmel JH, Pruyn JFA, Da Verwoerd C. An information system to support the care for head and neck cancer patients. *Supportive care in cancer : official journal of the Multinational Association of Supportive Care in Cancer* 2003; **11**: 452–9.

273 van den Brink JL, Moorman PW, Boer MF, Pruyn JFA, Da Verwoerd C, van Bemmel JH. Involving the patient: a prospective study on use, appreciation and effectiveness of an information system in head and neck cancer care. *INTERNATIONAL JOURNAL OF MEDICAL INFORMATICS* 2005; **74**: 839–49.

274 van den Brink JL, Moorman PW, Boer MF, *et al.* Impact on quality of life of a telemedicine system supporting head and neck cancer patients: a controlled trial during the postoperative period at home. *Journal of the American Medical Informatics Association* 2007; **14**: 198–205.

275 van der Hout A, van Uden-Kraan CF, Holtmaat K, *et al.* Role of eHealth application Oncokompas in supporting self-management of symptoms and health-related quality of life in cancer survivors: a randomised, controlled trial. *The Lancet Oncology* 2020; **21**: 80–94.

276 van Egdom LSE, Lagendijk M, van der Kemp MH, *et al.* Implementation of value based breast cancer care. *European Journal of Surgical Oncology* 2019; **45**: 1163–70.

277 Velikova G, Absolom K, Hewison J, *et al.* Electronic self-reporting of adverse events for patients undergoing cancer treatment: the eRAPID research programme including two RCTs. *Programme Grants for Applied Research* 2022; published online Feb. DOI:10.3310/FDDE8516.

278 Verweij L, Ector G, Smit Y, *et al.* Effectiveness of digital care platform CMyLife for patients with chronic myeloid leukemia: results of a patient-preference trial. *BMC HEALTH SERVICES RESEARCH*; **23**. DOI:10.1186/s12913-023-09153-9.

279 Vickers AJ, Savage CJ, Shouery M, Eastham JA, Scardino PT, Basch EM. Validation study of a web-based assessment of functional recovery after radical prostatectomy. *Health and quality of life outcomes* 2010; **8**. DOI:10.1186/1477-7525-8-82.

280 Vickers A, Assel M, Hannon M, *et al.* A comparison of brief versus explicit descriptors for verbal rating scales: interrupted time series design. *HEALTH AND QUALITY OF LIFE OUTCOMES*; **21**. DOI:10.1186/s12955-023-02184-0.

281 Vickers AJ, Salz T, Basch E, *et al.* Electronic patient self-assessment and management (SAM): a novel framework for cancer survivorship. *BMC medical informatics and decision making* 2010; **10**: 34.

282 Wagner LI, Schink J, Bass M, *et al.* Bringing PROMIS to practice: brief and precise symptom screening in ambulatory cancer care. *Cancer* 2015; **121**: 927–34.

283 Walker MS, Hasan M, Yim YM, Yu E, Stepanski EJ, Schwartzberg LS. Retrospective study of the effect of disease progression on patient reported outcomes in HER-2 negative metastatic breast cancer patients. *Health and quality of life outcomes* 2011; **9**. DOI:10.1186/1477-7525-9-46.

284 Wall LR, Cartmill B, Ward EC, *et al.* "ScreenIT": computerized screening of swallowing, nutrition and distress in head and neck cancer patients during (chemo) radiotherapy. *Oral oncology* 2016; **54**: 47–53.

285 Wang TQ, Samuel JN, Brown MC, *et al.* Routine Surveillance of Chemotherapy Toxicities in Cancer Patients Using the Patient-Reported Outcomes Version of the Common Terminology Criteria for Adverse Events (PRO-CTCAE). *Oncology and therapy* 2018; **6**: 189–201.

286 Warrington L, Absolom K, Holch P, Gibson A, Clayton B, Velikova G. Online tool for monitoring adverse events in patients with cancer during treatment (eRAPID): field testing in a clinical setting. *BMJ open* 2019; **9**. DOI:10.1136/bmjopen-2018-025185.

287 Warsame R, Cook J, Fruth B, *et al.* A prospective, randomized trial of patient-reported outcome measures to drive management decisions in hematology and oncology. *Contemporary clinical trials communications* 2022; **29**: 100964.

288 Watson L, Delure A, Qi S, *et al.* Utilizing Patient Reported Outcome Measures (PROMs) in ambulatory oncology in Alberta: Digital reporting at the micro, meso and macro level. *Journal of patient-reported outcomes* 2021; **5**: 97.

289 Wheelock AE, Bock MA, Martin EL, *et al.* SIS.NET: A Randomized Controlled Trial Evaluating a Web-Based System for Symptom Management After Treatment of Breast Cancer. *Cancer* 2015; **121**: 893–9.

290 Whitaker TJ, Mayo CS, Ma DJ, *et al.* Data collection of patient outcomes: one institution's experience. *JOURNAL OF RADIATION RESEARCH* 2018; **59**: I19–24.

291 Whitehead L, Emery L, Kirk D, Twigg D, Brown D, Dewar J. Evaluation of a Remote Symptom Assessment and Management (SAM) System for People Receiving Adjuvant Chemotherapy for Breast or Colorectal Cancer: Mixed Methods Study. *JMIR cancer* 2020; **6**: e22825.

292 Wintner LM, Giesinger JM, Sztankay M, Bottomley A, Holzner B. Evaluating the use of the EORTC patient-reported outcome measures for improving inter-rater reliability of CTCAE ratings in a mixed population of cancer patients: study protocol for a randomized controlled trial. *Trials* 2020; **21**: 849.

293 Wintner LM, Giesinger JM, Zabernigg A, *et al.* Evaluation of electronic patient-reported outcome assessment with cancer patients in the hospital and at home. *BMC medical informatics and decision making* 2015; **15**: 1–10.

294 Wright AA, Raman N, Staples P, *et al.* The HOPE Pilot Study: Harnessing Patient-Reported Outcomes and Biometric Data to Enhance Cancer Care. *JCO clinical cancer informatics* 2018; **2**: 1–12.

295 Wright EP, Selby PJ, Crawford M, *et al.* Feasibility and compliance of automated measurement of quality of life in oncology practice. *Journal of clinical oncology : official journal of the American Society of Clinical Oncology* 2003; **21**: 374–82.

296 Wu AW, White SM, Blackford AL, *et al.* Improving an electronic system for measuring PROs in routine oncology practice. *Journal of cancer survivorship : research and practice* 2016; **10**: 573–82.

297 Wysham NG, Wolf SP, Samsa G, Abernethy AP, LeBlanc TW. Integration of Electronic Patient-Reported Outcomes Into Routine Cancer Care: An Analysis of Factors Affecting Data Completeness. *JCO clinical cancer informatics* 2017; **1**: 1–10.

298 Yang HC, Chung SH, Yoo JS, Park B, Kim MS, Lee JM. Evaluation of a Smart After-Care Program for Patients with Lung Cancer: A Prospective, Single-Arm Pilot Study. *Journal of chest surgery* 2022; **55**: 108–17.

299 Young-Afat DA, van Gils CH, Bruinvels DJ, *et al.* Patients' and health care providers' opinions on a supportive health app during breast cancer treatment: a qualitative evaluation. *JMIR cancer* 2016; **2**: e5334.

300 Zhang LY, Zhang XT, Shen L, Zhu D, Ma SL, Cong L. Efficiency of Electronic Health Record Assessment of Patient-Reported Outcomes After Cancer Immunotherapy A Randomized Clinical Trial. *JAMA NETWORK OPEN* 2022; **5**. DOI:10.1001/jamanetworkopen.2022.4427.

301 Zhang R, Burgess ER, Reddy MC, *et al.* Provider perspectives on the integration of patient-reported outcomes in an electronic health record. *JAMIA open* 2019; **2**: 73–80.

302 Zini EM, Lanzola G, Bossi P, Quaglini S. An Environment for Guideline-based Decision Support Systems for Outpatients Monitoring. *Methods of information in medicine* 2017; **56**: 283–93.

303 Zylla DM, Gilmore GE, Steele GL, *et al.* Collection of electronic patient-reported symptoms in patients with advanced cancer using Epic MyChart surveys. *Supportive care in cancer : official journal of the Multinational Association of Supportive Care in Cancer* 2020; **28**: 3153–63.
