# Supplementary figures and images for: Sustainability and Time Trends in Electronic Patient-Reported Outcome Assessment in Routine Cancer Care: Systematic Scoping Review and Follow-Up Survey
Source: J Med Internet Res. 2025 Apr 25;27:e69398. doi: 10.2196/69398 (PMC12064961; doi:10.2196/69398)

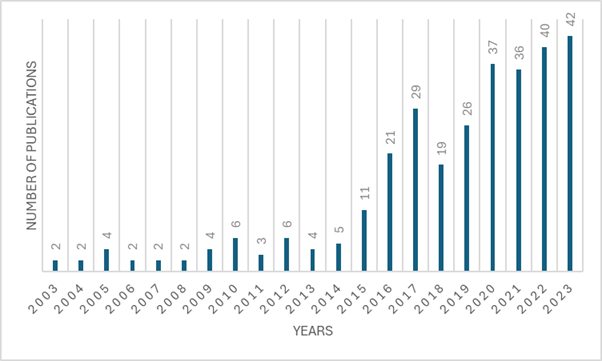

Supplement: Multimedia Appendix 3 [file jmir_v27i1e69398_app3.png]
